# Dual Blockade of LILRB1 and LILRB2 Enhances Antiviral Immune Responses in SIV Infection

**DOI:** 10.1002/advs.76557

**Published:** 2026-07-30

**Authors:** Florian Meurisse, Sixtine Coindre, Anne Wijkhuisen, Romain Marlin, Laure Fournier Le Ray, Juliette Pons, Melyssa Yaugel‐Novoa, Véronique Avettand‐Fenoel, Laurent Abi‐Rached, Anne‐Sophie Gallouet, Francis Relouzat, Mael Gourves, Asier Saez‐Cirion, Hisashi Arase, Gerard Zurawski, Sandra Zurawski, Nathalie Dereuddre‐Bosquet, Roger Le Grand, Stéphanie Simon, Olivier Lambotte, Benoit Favier

**Affiliations:** ^1^ Université Paris‐Saclay, Inserm, CEA; Immune Diseases, Microbiology and Innovative Therapies (IDMIT/UMRS1184) Fontenay‐aux‐Roses & Le Kremlin‐Bicêtre France; ^2^ SEPSIS Comprehensive Center‐IHU SEPSIS CEA France; ^3^ CEA INRAE Médicaments et Technologies pour la Santé (MTS) Université Paris‐Saclay Gif‐sur‐Yvette France; ^4^ Service de Virologie CHU d'Orléans Orléans France; ^5^ Université d'Orléans Orléans France; ^6^ SNC5039 CNRS Marseille France; ^7^ MEPHI Aix Marseille Université Marseille France; ^8^ IHU‐Méditerranée Infection Marseille France; ^9^ Viral Reservoirs and Immune Control Unit Institut Pasteur Université Paris Cité Paris France; ^10^ Department of Immunochemistry Research Institute for Microbial Diseases Osaka University Suita Osaka Japan; ^11^ Laboratory of Immunochemistry WPI Immunology Frontier Research Center Osaka University Suita Osaka Japan; ^12^ Center for Advanced Modalities and DDS Osaka University Suita Osaka Japan; ^13^ Center for Infectious Disease Education and Research Osaka University Suita Osaka Japan; ^14^ Baylor Institute for Immunology Research (BIIR) Dallas Texas USA; ^15^ Paris‐Saclay University Hospital Group Assistance Publique Hôpitaux de Paris Department of Internal Medicine and Clinical Immunology Bicêtre Hospital le Kremlin‐Bicêtre France

**Keywords:** blocking monoclonal antibody, costimulatory molecules, CD8+ T‐cell memory, ILT2, ILT4, immune checkpoint blockade, Leukocyte immunoglobulin‐like receptors (LILRs), myeloid immune checkpoints, preclinical model, Type III interferon

## Abstract

Restoring effective antiviral immunity remains a major challenge in HIV infection. Emerging immune checkpoint leukocyte immunoglobulin‐like receptor B1 (LILRB1) and LILRB2 have been proposed as therapeutic targets, yet their in vivo function remains undefined due to the lack of cross‐reactive blocking antibodies for relevant preclinical models. Here, we developed a dual‐specific blocking monoclonal antibody, mac20G10, targeting cynomolgus macaque LILRB1 and LILRB2 and assessed its immunomodulatory activity in an SIV model of infection. Pharmacodynamic analyses demonstrated that mac20G10 persisted in circulation and engaged target myeloid cells for up to 14 days without detectable adverse effects. A single administration prior to SIVmac251 infection enhanced early myeloid immune activation, characterized by increased frequencies of CD80^+^ plasmacytoid dendritic cells (pDCs) and CD80^+^ monocyte/macrophage subsets in blood and lymphoid tissues. These changes were accompanied by increased plasma levels of interferon lambda (IFN‐λ), IL‐8, and IL‐1RA during acute infection. Although viral replication remained unchanged, mac20G10 treatment promoted the development of SIV‐specific memory CD8^+^ T‐cell responses. Together, these data show that LILRB1/B2 blockade modulates early myeloid activation and is associated with enhanced SIV‐specific memory CD8+ T‐cell responses. These findings support further evaluation of LILRB1/B2 blockade as part of combination immunotherapeutic strategies in settings such as ART‐treated infection and analytical treatment interruption.

## Graphical abstract

Dual LILRB1/B2 blockade with mac20G10 reshapes myeloid activation during acute SIV infection by targeting LILRB1 and LILRB2 on myeloid cells. This treatment enhances CD80 expression on selected myeloid subsets and increases plasma IFN‐λ, IL‐8, and IL‐1RA. These early changes are associated with stronger SIV‐specific memory CD8+ T‐cell responses, highlighting a myeloid checkpoint pathway that shapes antiviral immunity.

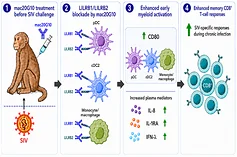

## 1 Introduction

Following the success of immunotherapy in cancer, strategies based on blocking immune checkpoints with monoclonal antibodies (mAb) are emerging as a promising treatment in HIV infection [1, 2, 3, 4, 5]. LILRB1 and LILRB2 are inhibitory receptors mainly expressed by monocytes, macrophages, and dendritic cells (DC) that play an important role in the regulation of immune responses, modulating the progression of infectious diseases and cancer [6, 7]. These receptors interact with classical and non‐classical MHC class I (MHC‐I) molecules, delivering inhibitory feedback signals that restrain myeloid cell activation [6, 7, 8, 9, 10, 11, 12]. Importantly, these LILRB1/B2‐mediated inhibitory signals can be reversed by blocking mAbs in vitro [13, 14]. However, investigation of their functions has been limited in vivo due to an absence of clear orthologs in murine models [7, 15].

HIV and SIV infections are characterized by an early dysregulation of myeloid immune responses that contributes to suboptimal adaptive responses and disease progression [16, 17, 18, 19]. We and others have previously demonstrated that expression of LILRB2 and its MHC‐I ligands is enhanced on myeloid cell subsets in early HIV and SIV infection [20, 21]. This enhancement of the LILRB2/MHC‐I inhibitory axis directly impairs the shaping of immune responses against HIV in vitro [22, 23, 24]. Furthermore, studies in people living with HIV exhibiting distinct progression profiles have shown that the strength of the LILRB2/MHC‐I interaction positively correlates with DC dysfunction and accelerated disease progression [25]. Elevated levels of soluble MHC‐I molecules in the plasma of people living with HIV further amplify this inhibitory pathway by engaging LILRB2 on monocytes and DC, thereby attenuating CD80 and CD86 up‐regulation and resulting in impairment of T cell priming [26, 27]. Although the contribution of LILRB1 in HIV infection remains less well defined, accumulating evidence suggests that multiple pathogens, including dengue virus, human cytomegalovirus and *Plasmodium falciparum*, exploit LILRB1 engagement to attenuate immune responses and promote persistence [28, 29, 30]. These convergent observations underscore the potential of targeting the LILRB1/MHC‐I interaction to reinvigorate antiviral immunity in chronic infections.

Despite compelling in vitro evidence supporting LILRB2 and LILRB1 as therapeutic targets in HIV infection, their roles remain to be characterized in vivo. To address this gap, we used the cynomolgus macaque (*Macaca fascicularis*) SIVmac251 infection model, which closely mirrors key features of human HIV infection, including a rapid peak in plasma viral load around day 14 post‐infection, followed by stabilization during the chronic phase. This well‐established model recapitulates major aspects of HIV pathogenesis in humans and provides a robust platform for investigating disease mechanisms in vivo. Notably, it has been instrumental in characterizing the dysregulation of myeloid immune cell subsets during early SIV infection and their downstream impact on adaptive immune responses [16, 17, 21, 31, 32, 33, 34, 35, 36].

Here, we report the development of a monoclonal antibody targeting cynomolgus macaque LILRB1 and LILRB2, designed to block their interaction with MHC‐I molecules. A single administration of the LILRB1/B2 blocker prior to SIV challenge enhanced early myeloid immune activation and elicited a specific cytokine signature, which was associated with improved induction of SIV‐specific CD8^+^ T cell responses during the chronic phase of infection. Together, these findings support further evaluation of LILRB1/B2 blockade as an immunomodulatory strategy in HIV/SIV infection.

## 2 Results

### 2.1 Development of a Blocking Monoclonal Antibody Targeting Cynomolgus Macaque LILRB1 and LILRB2

An anti‐LILRB1/B2 mAb was generated using cynomolgus macaque LILRB1 as an immunogen (Figure 1A). To this end, a soluble cynomolgus macaque LILRB1 extracellular region fused to the human IgG1 Fc portion was used to immunize mice. Hybridoma supernatants were initially screened for recognition of cynomolgus macaque LILRB1 (mafa‐LILRB1) and LILRB2 (mafa‐LILRB2) by ELISA and flow cytometry. Monoclonal antibodies of interest were then isolated and assessed for their specificity toward LILRB1 and LILRB2, as well as their ability to block interactions between LILRB1 or LILRB2 and Mafa‐A1*063 SIV^gag^ tetramers by flow cytometry. Clone 20G10 was selected based on its superior combined specificity for LILRB1/B2 and blocking activity. Flow cytometry analysis demonstrated that clone 20G10 recognizes mafa‐LILRB1 and mafa‐LILRB2, but not LILRB3 or LILRB4, nor LILRA1, LILRA2, or LILRA4 (Figure 1B). In addition, the 20G10 mAb efficiently blocked mafa‐LILRB2 binding to Mafa‐A1*063 SIV^gag^ tetramers (Figure 1C).

**FIGURE 1:**
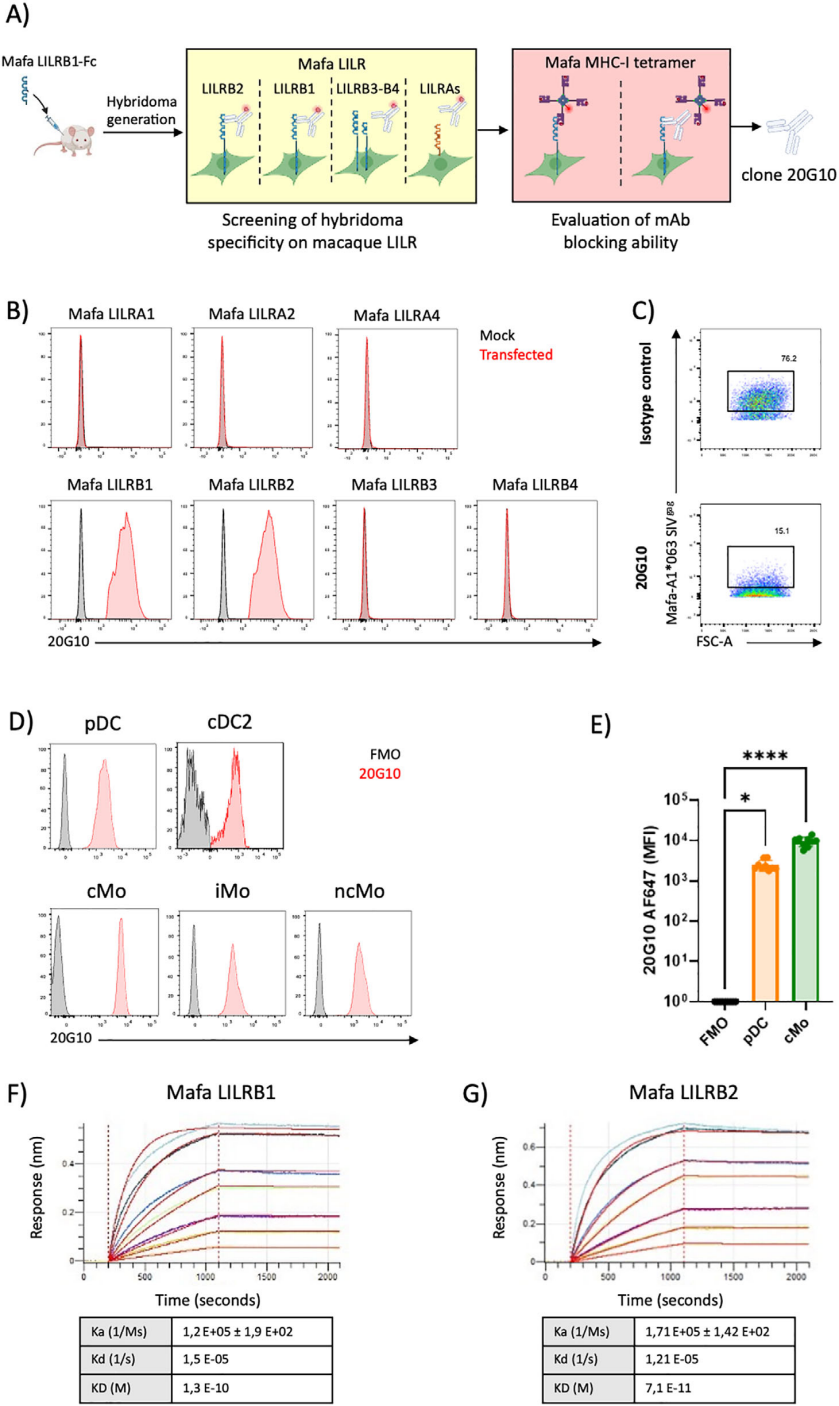
Development of an anti‐LILRB1 and LILRB2 dual‐blocking mAb (clone 20G10) specific to cynomolgus macaque. (A) Schematic overview of the strategy used to generate a dual‐blocking anti‐LILRB1/LILRB2 monoclonal antibody. (B) Determination of 20G10 mAb specificity using HEK293T expressing cynomolgus macaque receptor LILRA1, LILRA2, LILRA4, LILRB1, LILRB2, LILRB3 or LILRB4. Non‐transfected control cells are represented in grey. (C) 20G10 mAb blocking ability was assessed using a system of interaction between cynomolgus macaque LILRB2 expressed by HEK293T and cynomolgus macaque MHC‐I tetramers (Mafa‐A1*063) harboring a SIV^gag^ peptide. Stained cells were analyzed by flow cytometry. A significant reduction of LILRB2 binding to Mafa‐A1*063 tetramer demonstrated the blocking activity of the antibody. (D) Cell distribution of 20G10 mAb staining was assessed using flow cytometry. PBMCs from cynomolgus macaques were used to characterize 20G10 staining of pDC, cDC2, and monocyte subsets. cMo = classical monocytes, iMo = intermediate monocytes, and ncMo = non‐classical monocytes. 20G10 staining is represented in red and controls in grey. (E) Histograms representing MFI of pDC and cMo (*n* = 5) following staining with 20G10‐AF647; comparisons with negative controls were carried out using Dunn's statistical test; ^*^ = *p* < 0.05, ^****^ = *p* < 0.0001. (F, G) Binding parameters of 20G10 mAb to cynomolgus macaque LILRB1‐Fc (F) and LILRB2‐Fc (G) were assessed using Bio‐layer Interferometry. Sensorgrams and fitted curves are displayed, as well as the summary of the kinetic parameters. Cynomolgus macaque LILRB1‐Fc and LILRB2‐Fc were tested at seven concentrations ranging from 50 to 0.78nM in twofold serial dilutions, represented by different colours in the sensorgram.

Consistent with previous human studies, staining of cynomolgus macaque PBMCs with fluorescent 20G10 mAb confirmed that LILRB1 and LILRB2 are predominantly expressed by pDCs, conventional type 2 dendritic cells (cDC2), and monocyte subsets (Figure 1D,E; Figure S1). The 20G10 mAb also cross‐reacted with monocytes isolated from rhesus macaques (*Macaca mulatta*), another preclinical model widely used to investigate HIV pathogenesis (Figure S2).

The affinities of the 20G10 mAb for cynomolgus macaque LILRB1‐Fc and LILRB2‐Fc were measured by interferometry. High‐affinity binding was observed for both inhibitory receptors (K_D_ = 1.3 × 10^−10^ M and K_D_ = 7.1 × 10^−11^ M, respectively) (Figure 1F,G).

Overall, these data indicate that 20G10 specifically recognizes and blocks cynomolgus macaque LILRB1/B2 on myeloid cells in vitro, highlighting its potential as an immunotherapeutic candidate.

### 2.2 Humanization and Optimization of the 20G10 mAb for In Vivo Use

Since mouse mAb can be immunogenic in primates, the mAb clone 20G10 was modified by replacing mouse constant fragments with human IgG1 (ch20G10). The LALA mutations were introduced to minimize Fcγ receptor engagement and reduce the likelihood of Fc‐mediated leukocyte depletion or activation, whereas the YTE mutations were included to extend antibody half‐life in vivo [37] (Figure 2A). Next, variable regions of ch20G10 were humanized to a degree of 87.8% (hu20G10). Finally, human Fc IgG1 regions were replaced by cynomolgus macaque constant Fc IgG1 regions (including the LALA and YTE mutations), in order to increase homology with cynomolgus macaque physiology (mac20G10). The effects of these modifications on K_D_ were assessed for cynomolgus macaque LILRB1 by interferometry. Affinity was not altered by the combined series of modifications, with optimized mac20G10 possessing a similar K_D_ to the parental 20G10 isoform (Figure 2B). Apparent K_D_ values for cynomolgus macaque LILRB1 and LILRB2 were also measured by flow cytometry (Figure 2C,D). Slight decreases in affinity of mac20G10 for cynomolgus macaque LILRB1 or LILRB2 were observed, compared to ch20G10 (0.5913–2.075 µg/mL and 0.8779–2.699 µg/mL, respectively).

**FIGURE 2:**
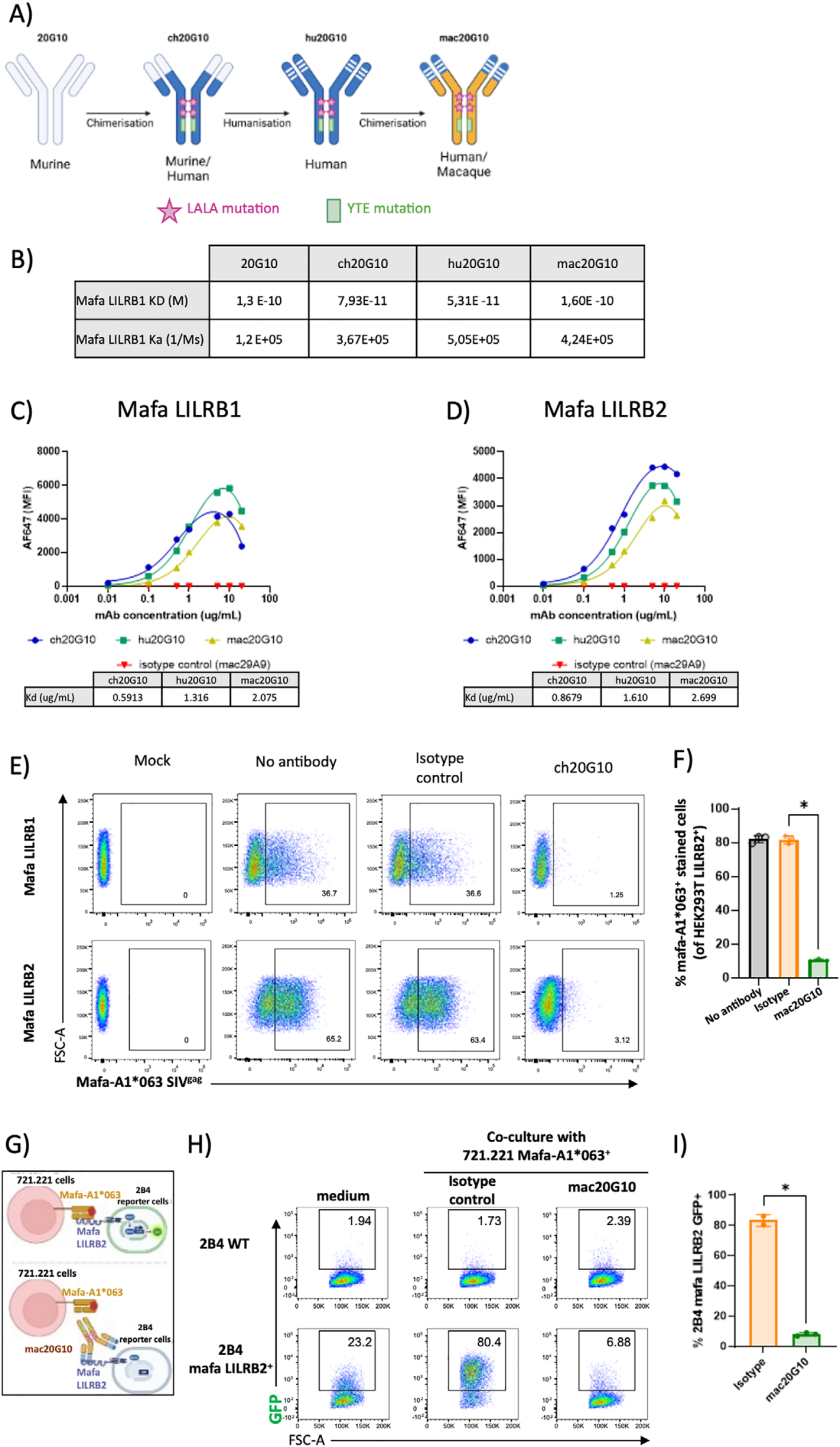
Optimization of anti‐LILRB1/B2 blocking antibody for in vivo use. (A) Schematic representation of the strategy used to optimize 20G10 mAb for cynomolgus macaque studies. The LALA mutation reduces the risks of adverse events by minimizing Fc receptor binding, while the YTE mutation enhances the antibody's half‐life in vivo by increasing affinity for FcRn. (B) Summary of KD and Ka of 20G10, ch20G10, hu20G10, and mac20G10 for cynomolgus macaque LILRB1‐Fc. KD was assessed using Bio‐layer Interferometry. (C, D) Apparent dissociation constants (Kd) of the different antibody variants determined by flow cytometry. HEK293T expressing either Mafa‐LILRB1 or Mafa‐LILRB2 was incubated with various concentrations of ch20G10, hu20G10, or mac20G10 to evaluate binding affinities to cynomolgus macaque LILRB1 (C) or LILRB2 (D). The mAb mac29A9 was used as a control. (E) ch20G10 mAb blocking ability was assessed using a system of interaction between cynomolgus macaque LILRB1 or LILRB2 expressed by HEK293T and cynomolgus macaque MHC‐I tetramers (Mafa‐A1*063) harboring a SIV^gag^ peptide. Stained cells were analyzed by flow cytometry. A significant reduction in tetramer binding demonstrates the efficacy of the antibody. (F) Histograms showing the frequency of HEK293T LILRB2^+^ cells stained with Mafa‐A1*063^+^ tetramers following the blocking assay performed in the absence of antibody (n = 3) or in the presence of either an isotype control (*n* = 3) or mac20G10 (*n* = 3) mAb. Comparisons with the isotype control were carried out using Dunn's statistical test; ^*^ = *p* < 0.05. (G) Schematic representation of the coculture reporter cell system assay. (H) mac20G10 mAb blocks the interaction of LILRB2 and MHC‐I in a co‐culture reporter cell system. The GFP signal is representative of a productive interaction between the expressed receptor and a ligand. (I) Bar graph showing the frequency of GFP+ 2B4 mafa‐LILRB2^+^ reporter cells cultured with medium alone (*n* = 2) or cocultured with 721.221‐Mafa‐A1*063 cells in the presence of isotype control (*n* = 2) or mac20G10 (*n* = 3). Comparisons with isotype controls were carried out using the Wilcoxon rank‐sum test; ^*^ = *p* < 0.05.

Blocking assays demonstrated efficient ch20G10‐mediated inhibition of LILRB1 or LILRB2 binding to the Mafa‐A1*063 SIV^gag^ tetramer (36.6% vs. 1.25% for LILRB1 and 63.4% vs. 3.12% for LILRB2; isotype control vs. ch20G10) (Figure 2E). This blocking capacity was preserved following antibody optimization (medians at 83.1% and 82.2% vs. 10.2%; without mAb and isotype vs. mac20G10, *p* = 0.049) (Figure 2F). The blocking activity of mac20G10 was further assessed using a coculture assay between NFAT‐GFP reporter cells expressing LILRB2, which produce GFP upon recognition of Mafa‐A1*063 presented by 721.221 cells (Figure 2G). Coculture resulted in robust GFP expression, reflecting productive receptor‐ligand interaction, and this signal was effectively abrogated by preincubation with mac20G10 but not with the isotype control. Importantly, mac20G10 displayed no agonistic activity, as no GFP expression was detected in reporter cells cultured with mac20G10 (Figure 2H,I).

Overall, these data indicate that the optimized mac20G10 is endowed with high affinity and blocking properties well suited for testing therapeutic applications.

### 2.3 Blocking LILRB1/B2 mAb Binds to Target Myeloid Immune Cells Without Inducing Adverse Events in Cynomolgus Macaques

Either ch20G10 or mac20G10 was administered to groups of healthy cynomolgus macaques, and pharmacodynamic parameters were characterized (Figure 3A). The persistence of circulating mAb in plasma, evaluated by ELISA, showed that ch20G10 and mac20G10 concentrations remained elevated for at least 14 days (Figure 3B). In parallel, the interaction between mAb and myeloid targets was monitored using a flow cytometry‐based competition assay, as previously described [38] (Figure 3C,D). In this assay, a reduced normalized AF647 signal indicates receptor occupancy by the administered antibody, whereas recovery of the signal reflects loss of target engagement over time. Both ch20G10 and mac20G10 remained bound to LILRB1/B2 on pDC for at least 14 days (Figure 3E,F). Accordingly, a strong correlation was observed between plasma persistence of the mAb and pDC binding (*r* = −0.7408, *p* = 0.000099) (Figure S3A). Similarly, anti‐LILRB1/B2 mAb remained bound to the surface of cDC2 and monocyte subsets for at least 14 days (Figure 3G,H). Furthermore, competition assays performed on pDC and cDC2 subsets isolated from axillary lymph node (LN) biopsies of *n =* 2 cynomolgus macaques treated with ch20G10 seven days earlier demonstrated that the mAb remained bound to both cell populations within the LN at day 7 post‐injection (Figure 3I).

**FIGURE 3:**
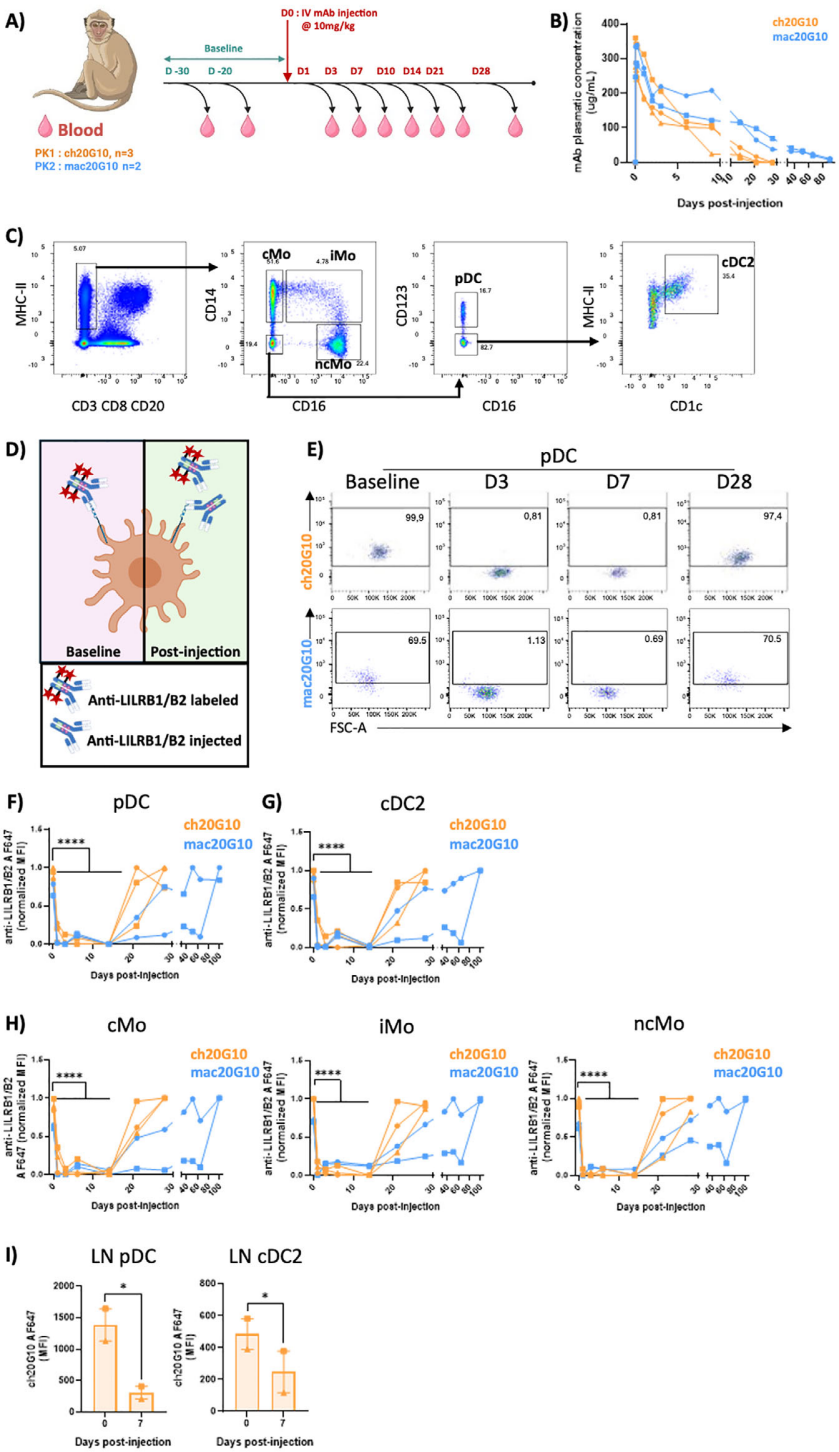
Pharmacodynamics of ch20G10 and mac20G10 in cynomolgus macaques. (A) Schematic representation of the pharmacodynamic studies schedule. Two groups were constituted. The first group received mAb ch20G10 (*n* = 3, 10 mg/kg, orange) and the second group received mAb mac20G10 (*n* = 2, 10 mg/kg, blue). (B) Plasma levels of ch20G10 (orange) and mac20G10 (blue) were monitored throughout the studies by ELISA. (C) Flow cytometry strategy used to monitor mAb binding to cMo = classical monocytes, iMo = intermediate monocytes, and ncMo = non‐classical monocytes, pDCs, and cDC2 during the pharmacodynamic studies. (D) Schematic representation of an ex vivo competition assay performed by flow cytometry to follow antibody persistence at the surface of myeloid cell subsets. (E) Representative flow cytometry dot plots of competition staining experiments of pDC during the follow‐up of the animals at baseline, D3, D7, and D28 post‐administration of ch20G10 or mac20G10. (F–H) Surface persistence of mAb on pDC, cDC2, and monocyte subsets was monitored by a competition staining assay. Values were normalized and compared to the baseline using mixed‐effect analysis, followed by a Dunn's multiple comparisons test as post‐hoc analysis; ^****^ = *p* < 0.0001. (I) Competition staining assays carried out by flow cytometry with ch20G10 on pDC and cDC2 from axillary lymph nodes (*n* = 2 individuals). Conditions were compared using a Mann‐Whitney statistical test; ^*^ = *p* < 0.05.

Safety monitoring during the pharmacodynamic studies was performed longitudinally after antibody administration and included clinical observations, body weight, temperature, hematological parameters, biochemical parameters, and changes in the major immune‐cell compartments. Administration of ch20G10 or mac20G10 had no detectable impact on behavior, body weight, temperature, or biochemical parameters, including total protein concentration, C‐reactive protein, gamma‐glutamyl transferase, alanine aminotransferase, aspartate aminotransferase, haptoglobin, alkaline phosphatase, creatinine, and urea. In addition, no depletion of major myeloid or lymphoid hematopoietic cell compartments was observed during the study, consistent with the LALA engineering strategy designed to limit Fcγ receptor engagement (Figure S3B–D).

Taken together, these data demonstrate that the recombinant ch20G10 and the optimized mac20G10 antibodies bind to LILRB1/B2 on myeloid cell subsets for at least two weeks following administration to cynomolgus macaques, without any detectable adverse events. These findings validate mac20G10 as a powerful tool for investigating the role of LILRB1/B2 in cynomolgus macaque preclinical models of human infectious diseases.

### 2.4 Effect of mac20G10 Treatment During SIVmac251 Infection

To ensure optimal tissue distribution and engagement of target immune cells prior to infection, cynomolgus macaques received a single dose of either anti‐LILRB1/B2 blocking antibody mac20G10 or the isotype control mac29A9, two days before SIVmac251 challenge. The 10 mg/kg dose was selected to ensure sustained systemic exposure and target engagement during the acute phase of infection and was subsequently confirmed to maintain LILRB1/B2 receptor occupancy on target myeloid subsets for approximately two weeks. The two groups were matched for sex and age, and all animals were negative for the MHC‐I H6 haplotype, given the potential impact of immunogenetic background on infection outcomes. Immune responses, plasma viral load, CD4+ T cell dynamics, and intact SIV DNA genomes were then monitored longitudinally in these animals (Figure 4A). No differences were observed between groups in plasma viral load kinetics, CD4+ T‐cell dynamics, or cell‐associated intact SIV DNA measured in PBMCs and secondary lymphoid organs, including lymph nodes and spleen (Figure 4B; Figures S4 and S5). However, mac20G10 remained detectable by ELISA in the plasma of treated animals for 10–14 days after SIV infection (corresponding to 12–16 days after mAb administration) (Figure 4C). The flow cytometry strategy enabled the simultaneous characterization of pDC, cDC1, cDC2, and monocyte subsets in peripheral blood (Figure S6A). Concordant with anti‐LILRB1/B2 kinetics measured in plasma (Figure 4C), competition assays indicated that mac20G10 remained bound to circulating pDC, cDC2, and classical monocytes (cMo) for 10–14 days post‐infection (Figure 4D). In this assay, reduced normalized AF647 signal indicated sustained target engagement by mac20G10 on circulating myeloid subsets. Similar results were observed for pDC and CD163^+^CD14^+^ macrophages isolated from peripheral LN using a dedicated flow cytometry strategy (Figure S6B), with binding detected on day 10 post‐infection (Figure 4E).

**FIGURE 4:**
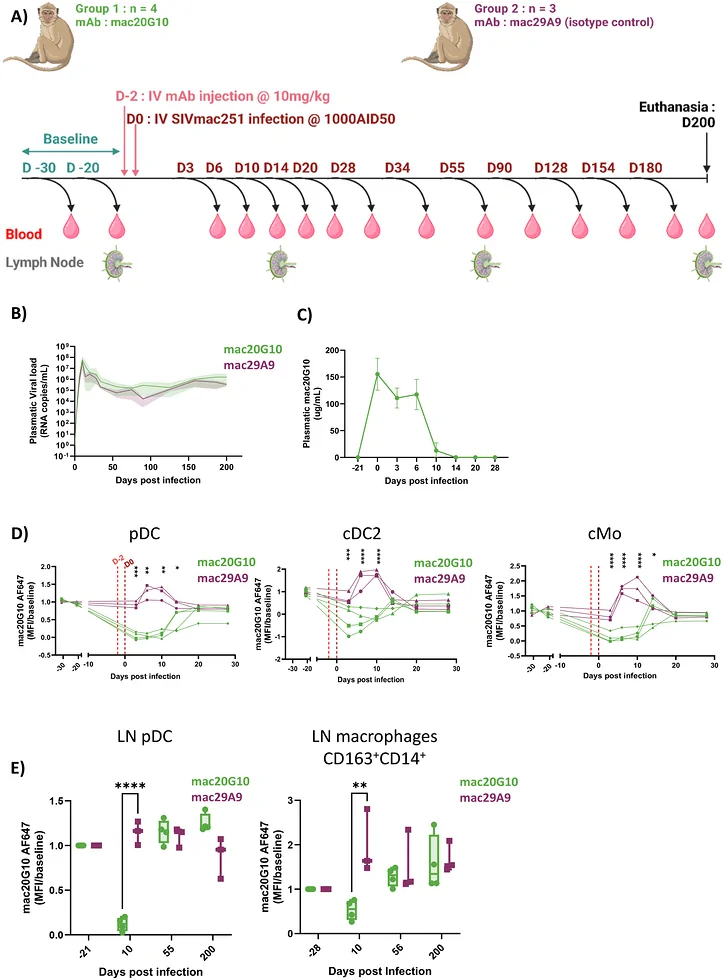
Longitudinal analysis of viral load and mac20G10 pharmacodynamics in SIV‐infected cynomolgus macaques. (A) Schematic representation of the study aiming to evaluate the impact of mac20G10 on immune responses in the SIV model. Two groups of cynomolgus macaques were used. One received the blocking anti‐LILRB1/B2 mac20G10 (*n* = 4, green) while the second received the isotype control mac29A9 (*n* = 3, purple), both before infection. Animals were followed for 200 days after infection. (B) Follow‐up of SIV plasma viral load post‐infection in both groups by RT‐qPCR. (C) Follow‐up of mac20G10 persistence in plasma by ELISA. (D) Surface persistence of mac20G10 on pDC, cDC2, and classical monocytes (cMo) monitored by competition staining assay. Normalized values are reported relative to baseline. The two groups were compared using a Kruskal‐Wallis test, followed by a Dunn's multiple comparisons test as post‐hoc analysis; ^*^ = *p* < 0.05, ^**^ = *p* < 0.01; ^***^ = *p* < 0.001; ^****^ = *p* < 0.0001. (E) Characterization of mac20G10 binding to pDC and macrophages (CD163^+^ CD14^+^) in lymph nodes by competition staining assay. Normalized values are reported relative to baseline. Data are shown as median with interquartile range and were compared using a Kruskal‐Wallis test, followed by a Dunn's multiple comparisons test as post‐hoc analysis; ^**^ = *p* < 0.01, ^****^ = *p* < 0.0001.

During the SIV infection study, hematological monitoring over the post‐challenge follow‐up period showed no detectable differences in target cell counts between control and mac20G10‐treated animals (Figure S7A). Consistent with previous findings, MHC‐I ligands were upregulated on cDC2 and monocyte subsets during early SIV infection (Figure S7B) [21].

These data indicate that a single administration of mac20G10 efficiently binds LILRB1/B2^+^ myeloid subsets for at least 10 days after SIV infection, but does not measurably affect plasma or tissue‐associated virological parameters, CD4+ T‐cell dynamics, or circulating immune‐cell composition.

### 2.5 mac20G10 Promotes CD80 Expression on pDCs and Monocytes/Macrophages and Increases Plasma IFN‐λ, IL‐8, and IL‐1RA in Early SIV Infection

To further evaluate the impact of LILRB1/B2 blockade on myeloid cell subsets during early cynomolgus macaque SIV infection, expression of key co‐stimulatory molecules CD80, CD86, and CD40 was assessed on myeloid populations from blood and LN by flow cytometry. Treatment with mac20G10 enhanced the frequency of CD80^+^ pDC at day 6 (medians: 16.3% vs. 4.7%; mac20G10 vs. control, *p* = 0.0049) and day 10 (medians: 58.6% vs. 45.9%; mac20G10 vs. control, *p* = 0.0015) post‐infection (Figure 5A,B). In addition, when compared to baseline, the proportions of CD80^+^ pDCs and cDC2 were significantly increased at day 6 post‐infection in the mac20G10‐treated group (pDC medians: mac20G10‐treated 0.5% to 16.3%, *p* = 0.0013, vs. control 0.2% to 4.7%; cDC2 medians: mac20G10‐treated 9.1% to 21.4%, *p* = 0.0020, vs. control 6.0% to 12.4%) (Figure 5B,C). At day 10 post‐infection, CD80 expression on cDC2 showed a non‐significant trend toward higher frequencies in mac20G10‐treated animals (medians: 56.9% vs. 41.7%; mac20G10 vs. control, *p* = 0.08), which should be interpreted cautiously given the limited group size.

**FIGURE 5:**
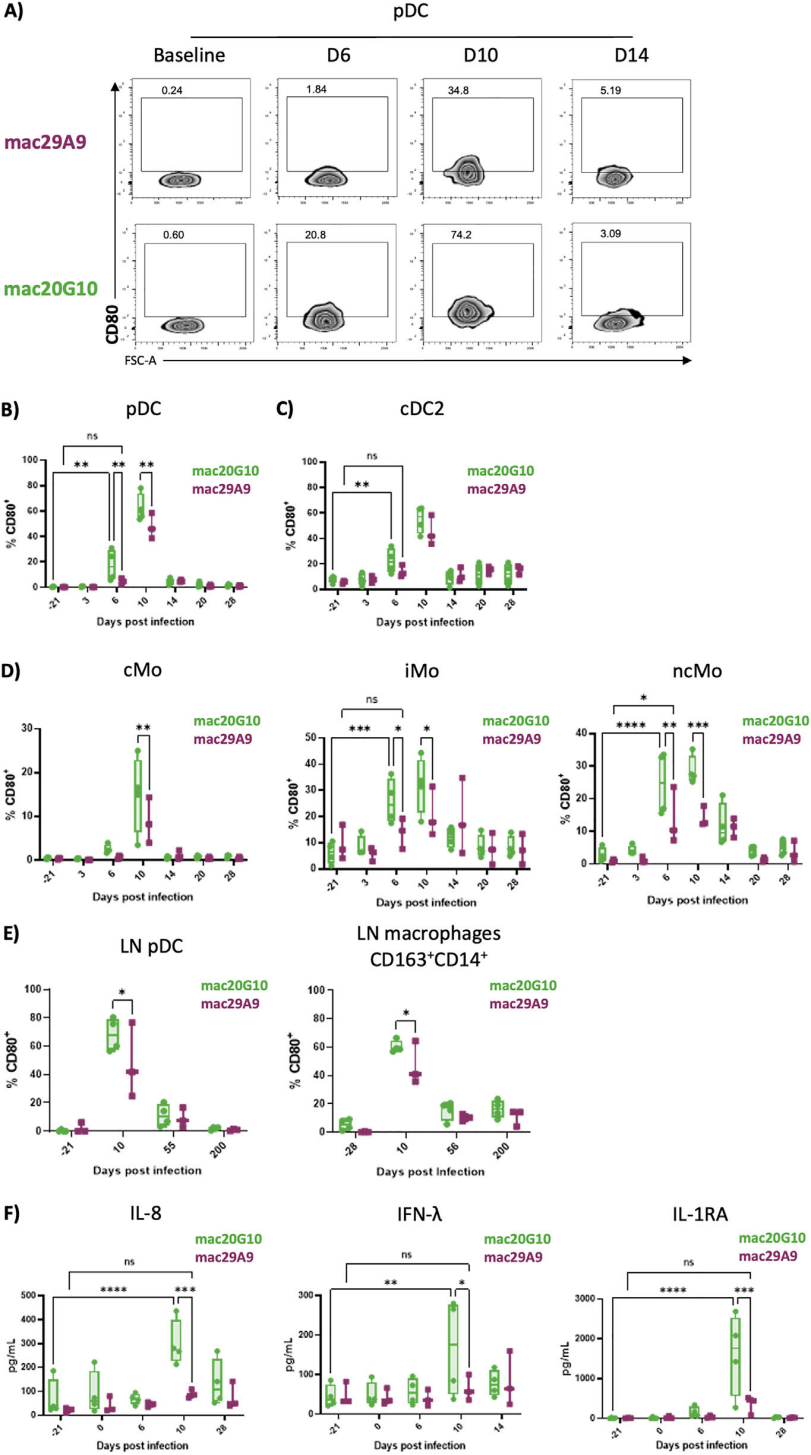
Follow‐up of the impact of mac20G10 on the frequencies of CD80^+^ pDCs, cDC2s, and monocytes/macrophages and on plasma cytokine production during early SIV infection. (A) Representative zebra plots of flow cytometry follow‐up of the CD80^+^ pDC subset during early SIV infection in cynomolgus macaques treated with mac20G10 or isotype control. (B) Follow‐up by flow cytometry of the CD80^+^ pDC population during early SIV infection in both groups. Data are represented as the median with interquartile range. Groups and time points were compared using a Kruskal‐Wallis test, followed by a Dunn's multiple comparisons test as post‐hoc analysis; ^**^ = *p* < 0.01. (C) Follow‐up of the CD80^+^ cDC2 population during early SIV infection by flow cytometry. Data are represented as the median with interquartile range. Groups and time points were compared using a Kruskal‐Wallis test, followed by a Dunn's multiple comparisons test as post‐hoc analysis; ^**^ = *p* < 0.01. (D) Follow‐up of CD80^+^ monocyte sub‐populations during early SIV infection by flow cytometry. cMo = classical monocytes, iMo = intermediate monocytes, and ncMo = non‐classical monocytes. Data are represented as the median with interquartile range. Groups and time‐points were compared using a Kruskal‐Wallis test, followed by a Dunn's multiple comparisons test as post‐hoc analysis; ^*^ = *p* < 0.05, ^**^ = *p* < 0.01, ^***^ = *p* < 0.001, ^****^ = *p* < 0.0001. (E) Follow‐up by flow cytometry of CD80^+^ pDC and macrophages (CD163^+^ CD14^+^) populations in the lymph node during SIV infection. Data are represented as the median with interquartile range. Groups were compared using a Kruskal‐Wallis test, followed by Dunn's statistical test; ^*^ = *p* < 0.05. (F) Follow‐up of plasma concentrations of IL‐8, IFN‐λ, and IL‐1RA during early SIV infection in cynomolgus macaques treated with mac20G10 or isotype control. Data are represented as the median with interquartile range. Time points and groups were compared using a Kruskal‐Wallis test, followed by a Šídák's multiple comparisons test as post‐hoc analysis; ^*^ = *p* < 0.05, ^**^ = *p* < 0.01, ^***^ = *p* < 0.001, ^****^ = *p* < 0.0001. The group treated with mac20G10 is represented in green (*n* = 4), and the mac29A9 isotype control group in purple (*n* = 3).

The frequencies of CD80^+^ monocyte subsets were higher at day 10 post‐SIV infection for the mac20G10‐treated group (cMo medians: 15.8% vs. 8.2%, *p* = 0.0089, iMo medians: 32.6% vs. 17.8%, *p* = 0.0326, and ncMo medians: 27.3% vs. 12.6%, *p* = 0.0002; mac20G10 vs. control) (Figure 5D). As observed for pDC, the proportion of CD80^+^ intermediate (iMo) and non‐classical (ncMo) monocytes had already increased by day 6 post‐infection after mac20G10 treatment.

Higher frequencies of CD80^+^ pDCs and macrophages were observed in mac20G10‐treated animals at day 10 after SIV infection (pDC medians: 67.9% vs. 41.7%, *p* = 0.0167, and macrophage medians: 58.9% vs. 40.8%, *p* = 0.0169; mac20G10 vs. control) (Figure 5E).

No significant impact of mac20G10 treatment was observed via analysis of CD40 and CD86 expression on pDC, cDC2, or monocyte subsets (Figure S8). In addition, mac20G10 treatment did not detectably affect cDC1 cell counts, MHC‐I expression, or co‐stimulatory molecule expression during early SIV infection (Figure S9).

Because LILRB1 can be expressed by NK‐cell subsets, we performed an exploratory phenotypic analysis and observed increased frequencies of MHC‐II+ CD16+ NK cells in mac20G10‐treated animals at day 10 post‐infection (medians: 4.69% vs. 1.56%, *p* = 0.0079; mac20G10 vs. control) (Figure S10). However, this analysis was limited to phenotypic markers, and NK‐cell cytotoxicity and cytokine production were not assessed.

Plasma cytokine concentrations were also analyzed during SIV infection. For isotype‐treated SIV‐infected animals, the cytokine expression profile was similar to the profiles previously observed in untreated SIV infection [39]. However, plasma levels of IL‐8, IFN‐λ and IL‐1RA were increased at day 10 post‐infection in animals treated with mac20G10 (IFN‐λ medians: 175.3 vs. 56.6 pg/mL, *p* = 0.0197; IL‐8 medians: 274.1 vs. 85.5 pg/mL, *p* = 0.0003, and IL‐1RA medians: 1754.6 vs. 445.3 pg/mL, *p* = 0.0001; mac20G10 vs. control) (Figure 5F). No difference was observed for other soluble immune mediators between the two groups (Figures S11 and S12).

Taken together, these results indicate that a single administration of mac20G10 induces a higher frequency of CD80^+^ myeloid cells in blood and LN, accompanied by concomitant increases in a specific set of cytokines during early SIV infection.

### 2.6 mac20G10 Administration Enhances the Development of SIV^gag^‐Specific CD8^+^ Memory T Cell Responses

To assess the impact of mac20G10 treatment on CD8^+^ T cell activation and IFN‐γ production during SIV infection, we stimulated cynomolgus macaque PBMCs ex vivo with a pool of SIV^gag^ peptides at baseline and 200 days post‐infection. The level of CD8^+^ T cell activation was then assessed by measuring the expression of the CD69 marker or the intracellular production of IFN‐γ by flow cytometry. Following stimulation, a higher frequency of CD69^+^ CD8^+^ T cells was observed in the chronic phase in the group that received mac20G10 (medians: 0.2%–4.1% for mac20G10, *p* = 0.0104; 0.2%–2.07% for control, *p* = 0.0948; baseline to chronic phase) (Figure 6A). Similarly, an enhancement of IFN‐γ production was observed in the mac20G10‐treated group (medians: 0%–1.2% for mac20G10, *p* = 0.0298; and 0%–0.51% for control, *p* = 0.0776; baseline to chronic phase) (Figure 6A).

**FIGURE 6:**
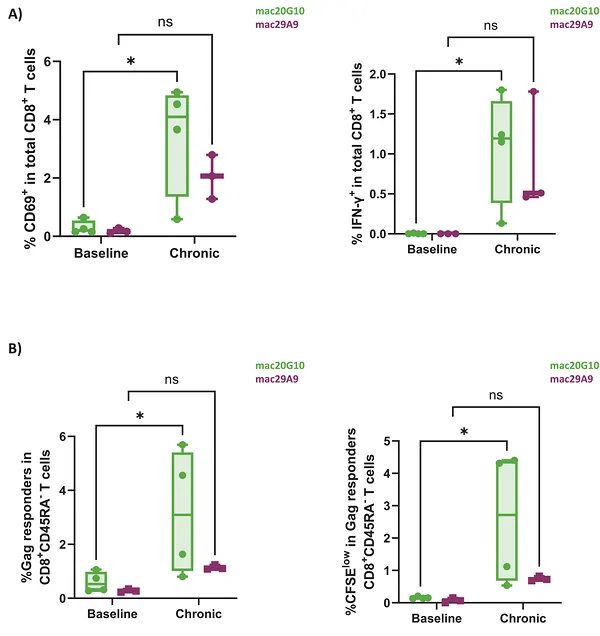
Evaluation of mac20G10 treatment effects on CD8^+^ T‐cell antiviral responses during the chronic phase of SIV infection. (A) Evaluation of specific CD8^+^ T cell responses to SIV^gag^ peptides ex vivo stimulation by measuring CD69 activation marker expression (left) and intracellular IFN‐γ production (right) by flow cytometry. Data are represented as the median with interquartile range. Time points and groups were compared using a Kruskal‐Wallis test, followed by an uncorrected Fisher's LSD test as post‐hoc analysis; ^*^ = *p* < 0.05, ^**^ = *p* < 0.01, ^***^ = *p* < 0.001. (B) Ex vivo evaluation of SIV‐specific memory CD8 T cell enrichment based on IFN‐γ and TNF‐α expressions (left) and ability to proliferate (right) over 6 days following SIV peptide stimulation. Data are represented as the median with interquartile range. Time points and groups were compared using a Kruskal‐Wallis test, followed by an uncorrected Fisher's LSD test as post‐hoc analysis; ^*^ = *p* < 0.05. mac20G10 treated group is represented in green (*n* = 4) and isotype control group in purple (*n* = 3). Chronic phase timepoint corresponds to day 200 (euthanasia).

Further analysis of the memory potential of CD8^+^ T cells demonstrated robust generation of SIV‐specific memory CD8^+^ T cells in the group treated with mac20G10, which was not significant in isotype‐treated animals (medians: 0.5%–3.1% for mac20G10, *p* = 0.0383; and 0.3%–1.1% for control, *p* = 0.4547; baseline to chronic phase). Furthermore, SIV‐specific CD45RA^−^ CD8^+^ T cells from mac20G10‐treated animals were characterized by cytokine production following expansion upon SIV‐peptide stimulation in contrast to the control group (medians: 0.1%–2.7% for mac20G10, *p* = 0.0268; and 0.1%–0.7% for control, *p* = 0.4923; baseline to chronic phase) (Figure 6B; Figure S13). Polyfunctional analysis based on IFN‐γ, TNF‐α, and CD107a expression after antigen‐driven expansion revealed a non‐significant trend toward increased polyfunctional SIV‐specific memory CD8+ T‐cell responses in mac20G10‐treated animals (Figure S13).

We also assessed post‐infection humoral responses over time. No significant differences were observed between mac20G10‐treated and control animals at any of the time points analyzed, indicating that LILRB1/B2 blockade did not measurably affect the magnitude of the humoral response in this experimental setting (Figure S14).

These results indicate that a single administration of mac20G10, just prior to SIV infection, is associated with enhanced CD8^+^ T cell memory responses against SIV during the course of SIV infection.

## 3 Discussion

Immune checkpoint blockade has revolutionized cancer immunotherapy but remains underexplored in chronic viral infections, where efforts have largely focused on reversing T cell exhaustion [40, 41].

Here, we provide in vivo evidence that blockade of the myeloid inhibitory receptors LILRB1 and LILRB2 modulates early myeloid activation during SIV infection and is associated with enhanced SIV‐specific memory CD8^+^ T‐cell responses. The engagement of LILRB1 and LILRB2 has been associated with dysfunctional immune responses and disease progression in HIV/SIV infection but also in other infectious diseases, yet in vivo functional evidence has been limited by the absence of suitable preclinical tools [6]. By developing a dual LILRB1/B2‐blocking antibody suitable for cynomolgus macaques, we were able to assess the immunomodulatory consequences of LILRB1/B2 blockade in a relevant macaque model of HIV infection.

Previous reports highlighted how alterations in co‐stimulatory molecule expression and enhanced LILRB2/MHC‐I inhibitory signaling were hallmarks of DC and monocyte/macrophage dysregulation in SIV and HIV infection that could lead to impaired immune responses [16, 20, 21, 22, 23, 24, 25, 26, 27, 42]. Analysis of the lymphoid tissues from people living with HIV and SIV‐infected macaques also underlined defective expression of co‐stimulatory molecules by DC and up‐regulation of LILRB2 [21, 43, 44]. Our data indicate that LILRB1/B2 blockade selectively enhances CD80 expression on targeted myeloid subsets during early SIV infection, without detectable modulation of CD86 or CD40. This pattern suggests restricted remodeling of selected co‐stimulatory pathways rather than broad myeloid maturation, consistent with the independent regulation of CD80, CD86, and CD40 reported during primary SIV infection [17, 42]. Mechanistically, LILRB1/B2 blockade may relieve ITIM‐dependent inhibitory signaling and lower the threshold for CD80 upregulation in myeloid cells exposed to acute inflammatory cues. Although increased CD80 may influence CD28‐ or CTLA‐4‐dependent T‐cell interactions, its functional consequences cannot be inferred from phenotypic data alone. Future co‐culture assays using sorted myeloid subsets and autologous T cells will be required to determine whether CD80 upregulation improves antigen presentation, antigen‐specific T‐cell priming, or T‐cell skewing.

Enhanced myeloid activation was accompanied by increased plasma IL‐8, IL‐1RA and IFN‐λ, indicating a selective cytokine signature after LILRB1/B2 blockade. Monocytes/macrophages and neutrophils may contribute to IL‐8 and IL‐1RA production, whereas pDCs represent a plausible source of IFN‐λ in the context of viral sensing [45, 46, 47]. However, other myeloid and epithelial cells may also contribute to IFN‐λ production depending on the tissue context. Nevertheless, because cytokines were measured in plasma, their cellular sources and functional consequences remain unresolved.

Because type I IFN production by pDCs is rapid and transient, our sampling schedule may not have fully captured short‐lived differences between groups. Plasma IFN‐α2 peaked at day 10 post‐infection, with no significant difference between mac20G10‐treated and control animals, and the next available time point was day 21. Thus, more frequent acute‐phase sampling, combined with ex vivo pDC stimulation assays, will be required to determine whether mac20G10 directly modulates pDC‐derived type I IFN responses in vivo.

We previously reported positive correlations between CD80 expression on DCs and CD8+ T‐cell activation in human elite HIV controllers [36]. In the present study, LILRB1/B2 blockade was associated with enhanced SIV‐specific CD8+ T‐cell responses during the chronic phase, supporting a model in which early myeloid modulation may influence downstream antigen‐specific CD8+ T‐cell immunity. However, the mechanisms linking CD80 upregulation to improved CD8+ T‐cell function remain to be defined and will require dedicated ex vivo functional assays. In addition, because our stimulation assay used optimal MHC class I‐restricted CD8+ T‐cell peptides, it was not designed to comprehensively assess SIV‐specific CD4+ T‐cell responses.

Despite the induction of measurable immunological effects, mac20G10 treatment did not reduce plasma viral load, alter CD4+ T‐cell dynamics, or decrease tissue‐associated intact SIV DNA. The absence of detectable virological control may reflect the small number of animals and the high‐dose intravenous SIVmac251 challenge, which induces rapid systemic replication and may exceed the capacity of early immune modulation to constrain viremia. Thus, enhanced immune responses do not necessarily translate into acute virological control, as previously reported for T cell‐targeted immune checkpoint blockade in SIV infection [38, 48, 49]. In addition, the available tissue analyses did not allow reliable discrimination between productively infected myeloid cells, infected T cells, and myeloid cells that had captured viral material. Future spatial viral RNA/DNA approaches combined with cell‐type markers will be helpful to resolve the cellular distribution of infection in this context.

The present study was performed during primary SIV infection, with a single pre‐infection administration of mac20G10 designed to characterize early myeloid dysregulation and confirm the biological activity of LILRB1/B2 blockade in vivo. Although this approach provided mechanistic insight into immune priming, its direct translational relevance remains limited because it does not model established infection, ART‐treated infection, or analytical treatment interruption. Therefore, the relevance of LILRB1/B2 blockade to HIV remission strategies remains inferential and will require dedicated evaluation in ART‐suppressed animals undergoing treatment interruption [50, 51]. In such settings, LILRB1/B2 blockade could be tested for its capacity to reverse pre‐existing myeloid dysfunction, enhance antiviral T‐cell responses, and contribute to immune‐mediated control of viral rebound. Because both the magnitude of viral rebound and the efficiency of antiviral effector responses are key determinants of post‐ART control, administration of mac20G10 around the time of ART cessation, potentially through repeated dosing before and after interruption, may help enhance antiviral immunity and improve the likelihood of post‐treatment control [52, 53]. However, given the multifactorial nature of immune dysfunction in SIV/HIV infection, targeting myeloid inhibitory pathways alone may be insufficient to achieve durable viral control. This supports the integration of LILRB1/B2 blockade into broader combinatorial immunomodulatory strategies, consistent with recent studies combining complementary immune‐targeting approaches [54].

Consistent with this rationale, immune checkpoint blockade strategies have been explored in SIV‐infected macaques, including CTLA‐4 and PD‐1 blockade [38, 48, 55, 56, 57, 58, 59]. These studies showed that checkpoint inhibition can enhance antiviral T‐cell responses, although effects on viral replication have been variable and context‐dependent. In particular, PD‐1 blockade has been associated with improved SIV‐specific CD8+ T‐cell responses in some settings, whereas CTLA‐4 blockade before infection increased viral replication at mucosal sites [38, 48, 56, 57, 58, 59]. These observations highlight both the potential and the complexity of immune checkpoint modulation in SIV/HIV infection. In this context, LILRB1/B2 blockade could represent a complementary strategy aimed at modulating myeloid inhibitory pathways upstream of T‐cell activation. However, repeated dosing would require dedicated evaluation of pharmacokinetics, safety, and anti‐drug antibody responses [60].

In summary, our study shows that dual blockade of the myeloid inhibitory receptors LILRB1 and LILRB2 induces measurable myeloid immune modulation during early SIV infection and is associated with enhanced SIV‐specific memory CD8+ T‐cell responses. Although mac20G10 treatment did not achieve detectable virological control in this model, these findings provide in vivo evidence that targeting myeloid inhibitory pathways can modulate antiviral immune responses. This supports further evaluation of LILRB1/B2 blockade in more clinically relevant settings, particularly ART‐treated infection and analytical treatment interruption, and as part of broader combination immunotherapeutic strategies.

### 3.1 Limitations of the Study

Our study has several limitations. First, the small number of animals, inherent to early‐stage non‐human primate studies, may have limited our ability to detect modest effects on viral replication, CD4+ T‐cell dynamics, or tissue‐associated SIV DNA. Second, the use of a high‐dose intravenous SIVmac251 challenge and a single pre‐infection administration of mac20G10 may have limited the capacity of early immune modulation to affect viral control. This experimental setting therefore provides biological proof of concept for LILRB1/B2 blockade but does not directly model established infection, ART‐treated infection, or analytical treatment interruption. Future studies using larger groups, more physiologically relevant mucosal challenge models, and settings of established or ART‐treated infection will be required to further evaluate the impact of LILRB1/B2 blockade on antiviral immunity and viral control.

## 4 Material and Methods

### 4.1 Ethics Statements

Cynomolgus and rhesus macaques were imported from Mauritius and housed in facilities at the Infectious Diseases Models and Innovative Therapies (IDMIT) center (CEA site in Fontenay‐aux‐Roses, France). Non‐human primate studies were approved by the ethics committee “Comité d'éthique en expérimentation animale” No. 44, and by the “*Ministère de l'Education Nationale, de l'Enseignement Supérieur et de la Recherche*” (France) (*Reference *: APAFIS#20525‐2019050616506478 and APAFIS #39765‐2022121215145877 v1). All animal treatments and procedures were performed according to French national regulations and, by association, European Union regulations under the direct supervision of national veterinary inspectors (*CEA accreditation No. D92‐032‐02*) (*European Directive 2010/63, recommendation No. 9*), in compliance with the Standards for Human Care and Use of Laboratory Animal Welfare (*OLAW, USA; under OLAW Assurance No. A5826‐01* and #F22‐00556).

### 4.2 Mice

Biozzi mice were bred at the animal care unit of the CEA (Gif‐sur‐Yvette, France). All experiments were performed in compliance with French and European regulations on the care of laboratory animals (European Community Directive 86/609, French Law 2001–486, 6 June 2001) and with the agreements of the Ethics Committee of the Commissariat à l'Energie Atomique (CEtEA ‘Comité d'Ethique en Expérimentation Animale’ no. 44) no. 12–026 and 15–055 delivered to S. S. by the French Veterinary Services and CEA agreement D‐91‐272‐106 from the Veterinary Inspection Department of Essonne (France).

### 4.3 Human Samples

Blood samples of healthy subjects were obtained from anonymous donors via the “*Etablissement Français du Sang*” (EFS).

### 4.4 Cell Lines, Cell Culture, and Transduction

The human MHC‐I deficient 721.221 cell line and murine 2B4 reporter cells were cultured at 37°C and 5% CO_2_ in RPMI 1640 GlutaMAX (Gibco) containing 10% fetal bovine serum (FBS) (Sigma–Aldrich) and 1% Penicillin‐Streptomycin (Gibco). Human 293T embryonic kidney (HEK293T) cells were cultured at 37°C and 5% CO_2_ in DMEM GlutaMAX (Gibco) complemented with 10% FBS and 1% Penicillin–Streptomycin (Gibco). Cells were periodically tested for mycoplasma contamination using the MycoAlert Mycoplasma Detection kit (Lonza).

HEK293T, 721.221, and 2B4 cell lines expressing either LILR or MHC‐I of interest were obtained through lentiviral transduction. Briefly, cells were concentrated to around 5 × 10^6^ cells/mL, and lentiviral particles were added with an MOI of 15. Suspensions were incubated at 37°C for 1 h with gentle mixing every 15min. At the end, medium was added in order to dilute cells to 0.25 × 10^6^ cells/mL. After 16 h of incubation, the medium was changed to fresh medium, and cells were plated in T25 flasks. After 4 days of culture, cells were stained with either anti‐HLA‐I (clone W6/32) or anti‐LILR in PBS + 0.5% BSA for 30min at 4°C. Then, cells were washed and sorted for the expression of LILRs or MHC‐I and GFP reporter gene using BD FACSAria Fusion cell Sorter (BD).

### 4.5 Cynomolgus Macaque LILR‐Fc Fusion Protein Production

The LILR‐Fc fusion proteins were produced using the LILRB1 cynomolgus macaque cDNA sequence (Reference XP_045236898.1). The cDNAs encoding the extracellular domain of the macaque LILRB1 or LILRB2 were fused to an hIgG1‐Fc by molecular biology. The plasmid constructions were transfected into ExpiHEK293T cells, and supernatants were harvested to purify LILRB1‐Fc or LILRB2‐Fc proteins by affinity chromatography.

### 4.6 LILRB1/B2 Monoclonal Antibody Generation, Optimization, and Production

Monoclonal antibodies (mAbs) were raised in Biozzi mice by immunizing with cynomolgus macaque LILRB1‐Fc (30 µg per injection) fusion protein. Mice eliciting the highest anti‐LILRB1 antibody response were given an intravenous boost injection 3 days before being sacrificed for splenic B cell fusion, according to Köhler and Milstein [61]. Hybridoma culture supernatants were screened for antibody production, specificity for cynomolgus macaque LILRB1 and LILRB2, and blocking capacity by enzyme immunoassay, flow cytometry, or both methods. Selected hybridomas were subsequently cloned by limiting dilution. Monoclonal antibodies were produced in hybridoma supernatants and further purified using an AKTA protein purifier. Purity of monoclonal antibodies was assessed by SDS‐PAGE in reducing and non‐reducing conditions using an Agilent 2100 Bioanalyser. Endotoxin levels in purified mAbs were determined by Pierce LAL Chromogenic Endotoxin Quantitation Kit (Thermo Fisher Scientific) according to the manufacturer's protocol.

cDNA corresponding to variable regions of selected hybridoma clone 20G10 was amplified by RT‐PCR and sequenced by the Sanger method. ch20G10 was obtained by grafting cDNA sequences of variable regions into a vector containing human IgG1 Fc constant regions harboring LALA (L234A, L235A) and YTE (M252Y, S254T, T256E) mutations.

The humanization of 20G10 variable regions was performed by deep mutation scanning (DMS) and yeast surface display (YSD) as described previously [62]. Humanized 20G10 variants were produced and tested in vitro before the selection of the best candidate.

Mac20G10 was obtained by grafting the selected humanized 20G10 sequence to cynomolgus macaque IgG1 constant chain sequences, including LALA and YTE mutations. The isotype control mac29A9 was generated using variable region sequences specific to a hapten.

The monoclonal antibodies ch20G10 and mac20G10 were produced on a large‐scale in ExpiCHO cells. The mAbs were then purified from supernatant using affinity chromatography with protein A in low‐endotoxin conditions. Quality controls were assessed using SDS‐PAGE, spectrophotometry, and SEC‐HPLC. The protein sequences of the antibody variants are included in a related patent application (EP 3 981 789).

### 4.7 Immunoenzymatic Screening of Anti‐LILRB1 and ‐LILRB2 Hybridoma Supernatants

Enzyme immunoassays (EIA) were performed by transferring 50 µL of diluted hybridoma culture supernatants into 96‐well, flat‐bottom microplates (MaxiSorp immunoplate, Nunc) coated with goat anti‐mouse immunoglobulin antibody (Jackson ImmunoResearch) and incubated overnight at 4°C. After washing, biotinylated mLILRB1‐Fc or mLILRB2‐Fc fusion proteins (50 ng/mL) were added (100 µL per well), and plates were reacted for 1 h at room temperature (RT). Plates were washed and reacted for 30 min at RT with 100 µL per well of 1 EU/mL of acetylcholinesterase (AChE)‐labeled streptavidin. After several washes, AChE activity was revealed by Ellman's colorimetric method [63] by measuring absorbance at 414 nm after 1 h. All reagents were diluted in EIA buffer (0.1 M phosphate buffer [pH 7.4] containing 0.15 M NaCl, 0.1% BSA, and 0.01% sodium azide). Plates coated with proteins were saturated in EIA buffer (18 h at 4°C) and washed with washing buffer (0.1 M potassium phosphate [pH 7.4] containing 0.05% Tween 20).

### 4.8 Affinity Determination of mAbs

The affinities of mAbs for the different macaque LILRs‐Fc fusion proteins were determined by Bio‐layer Interferometry using the ForteBio system (Pall Laboratory). mAbs prepared at 10µg/mL in EIA buffer + 0.02% Tween 20 (Sigma–Aldrich) were dispensed in a 96‐well microplate. In another well, LILR‐Fc fusion proteins were each dispensed at 8 titrated concentrations. A glycine (Sigma–Aldrich, pH [1.4]) regeneration solution and EIA buffer + 0.02% Tween 20 for baseline stabilization and neutralization were also prepared. The plate was agitated at 1000 rpm over the entire course of the experiment. Prior to the binding measurements, the anti‐mouse Fc (AMC) sensor tips were hydrated in EIA buffer + 0.02% Tween 20. The sensor tips were then transferred to the EIA buffer + 0.02% Tween 20 for the baseline, after mAb‐containing wells for the 300 s loading step. After the baseline step in EIA buffer + 0.02% Tween 20 for 60 s, the binding kinetics were measured by dipping the mAb‐coated sensors into the wells containing LILR‐Fc fusion protein at varying concentrations. The binding interactions were monitored over a 900 s association period and followed by a 900 s dissociation period in the wells containing EIA buffer +0.02% Tween 20. The AMC sensor tips were regenerated with wells containing glycine [pH 1.4] and neutralized in the EIA buffer + 0.02% Tween 20 between each binding cycle. The equilibrium dissociation constant (KD) was calculated using the ratio between the dissociation rate constant (koff) and the association rate constant (kon), obtained with global Langmuir 1:1 fit (Octet Data Analysis software, vHT.10).

Apparent Kd was also determined using flow cytometry. Briefly, HEK293T cells expressing macaque LILRB1 or LILRB2 were plated at 10^5^ cells per well in a 96 U‐well plate and incubated with either anti‐LILRB1/B2 mAb, isotype control, or without antibody for 1.5 h at 4°C, with periodic agitation. Then, cells were washed twice in PBS 0.5% BSA and stained with polyclonal anti‐human IgG‐AF647 antibodies (Jackson ImmunoResearch) for 30 min. Finally, cells were centrifuged at 200g for 7min and resuspended in Fixation buffer (Biolegend) diluted at 1:4 in 1X PBS and then analyzed by flow cytometry.

### 4.9 Cross‐Reactivity Assay

HEK293T cells expressing macaque LILRs were used to assess the cross‐reactivity of anti‐LILRB1/B2 mAb. The different macaque LILRs contained an N‐terminal DYKDDDDK Flag tag to check their expression at the cell surface by flow cytometry with an anti‐Flag APC (Miltenyi). Cells were incubated with anti‐LILRB1/B2 mAb or isotype control for 30 min at 4°C. Then, cells were centrifuged at 200 g for 7 min and resuspended in Fixation buffer (Biolegend) diluted at 1:4 in 1X PBS.

### 4.10 Blocking Assay

HEK293T cells expressing macaque LILRB1 or LILRB2 were plated at 10^5^ cells per well in a 96‐well plate and washed once. Cells were first incubated for 20 min at RT with 10 µg/mL of either anti‐LILRB1/B2 mAb, isotype control, or no antibody. Then, fluorescent tetramers Mafa‐A1*063‐PE carrying SIV^gag^ peptide (Clinisciences) were added for 30min at 4°C. Blocking ability was determined by flow cytometry, based on PE signal detection.

### 4.11 Reporter Assay

721.221‐Mafa‐A1*063 cells were co‐incubated with either 2B4 reporter cells expressing macaque LILRB2 fusion protein or 2B4 parental cells at a 1:1 ratio in a 96 well U‐bottom plate. Then, cells were incubated at 37°C and 5% CO2 for 24 h in the presence of anti‐LILRB1/B2 (20 µg/mL) or isotype control (20 µg/mL). At the end, cells were washed and stained with anti‐human HLA‐I (W6/32, Biolegend) and anti‐human CD45 (HI30, BD) for 30min at 4°C to identify each cell line, and GFP production was quantified by flow cytometry.

### 4.12 Pharmacodynamic Studies

Two pharmacodynamic studies were carried out with either ch20G10 (*n* = 3) or mac20G10 (*n* = 2) versions of anti‐LILRB1/B2 blocking mAb. Antibodies were infused intravenously at 10mg/kg body weight on day 0. Blood samples were collected before mAb administration and up to 4 months following injection.

### 4.13 Antibody Administration and SIV Infection

Seven female macaques were included in the study. They were 3 years old at inclusion. The first group (*n* = 4) and the second group (*n* = 3) were infused with either mac20G10 or mac29A9 isotype control, respectively. Antibodies were administered intravenously at 10mg/kg of body weight 48 h before infection. Both groups were infected intravenously with 1,000 animal infectious dose 50% (1000 AID_50_) of SIVmac251 on day 0. Macaques were periodically sampled, then euthanized 6 months after infection. Animals were negative for the MHC‐I H6 haplotype in order to prevent natural control of SIVmac251 viral infection.

### 4.14 Sample Collection and Processing

Blood for complete blood cell count, phenotyping, and plasma assays was collected in EDTA tubes (Vacutainer, BD, USA). Blood for functional assays was collected in Cell Preparation Tube (CPT) lithium‐heparin tubes (Vacutainer, BD, USA). Blood for serum collection was collected in dry tubes (Vacutainer, BD, USA). Peripheral lymph nodes (LN) were harvested from the right and left inguinal and axillary nodes in a clockwise direction. All procedures were performed under general anesthesia by intramuscular injection of ketamine (Imalgene 1000, 10 mg/kg) and medetomidine (Domitor, 0.5 mg/kg).

EDTA and dry tubes were centrifuged at 2100 g for 10 min, at room temperature, to obtain, respectively, plasma and serum. Plasma was used to follow circulating antibody concentration by an in‐house ELISA, anti‐SIV antibodies, cytokine concentration by Luminex or Legendplex, and viral load by RT‐qPCR. The buffy coat was diluted 1:1 in PBS 1X (Gibco). After sampling 100 µL for whole blood staining, the cell suspension was supplemented with homemade hypotonic red blood cell lysis buffer at a ratio of 1:15. Whole blood leukocytes obtained were recovered by centrifugation at 200g for 10mins and cells were resuspended in PBS 1X + 0.5% BSA (Sigma–Aldrich). Cell count was assessed using a Vi‐Cell XR automated hemocytometer (Beckman Coulter).

LN samples were divided in half with a scalpel before mechanical dissociation of single cells, using gentle pressure applied with a sterile syringe plunger on a 70 µm nylon filter. The filter was periodically washed with fresh RPMI 1640 GlutaMAX containing 10% FBS and 1% Penicillin–Streptomycin. Finally, LN cells were recovered by centrifugation at 200g (4°C) for 10 mins. Final cell suspensions were resuspended in PBS 1X + 0.5% BSA.

### 4.15 Competition Assay

Whole blood cells were stained with anti‐LILRB1/B2 mAb labeled with AF647 (Antibody Labeling Kits Alexa Fluor 647, Thermo Fisher) for 30 min at 4°C. Cells were analyzed using flow cytometry after washes and fixation. The absence of signal post‐injection indicates that macaque LILRB1/B2 receptors are saturated by the antibody administered.

### 4.16 Anti‐LILRB1/B2 mAb Distribution and Reactivity Cross‐Macaque

Staining of immune cell subsets by 20G10 from cynomolgus or rhesus macaques was performed by incubating whole blood cells with appropriate antibody cocktails for 30min at 4°C. Cells were analyzed using flow cytometry after washes and fixation. Antibodies are described in Table S1.

### 4.17 Immune Cell Phenotyping and Functional Assays

Antibody cocktails were prepared in PBS 1X + 0.5% BSA, using previously calculated saturating concentrations. For each sample, whole blood cells were labeled with the appropriate antibody cocktail. For the competition assay, anti‐LILRB1/B2 mAb labeled with AF647 was included. Whole blood cells were pre‐incubated with the corresponding antibody cocktail for 30min at 4°C. Subsequently, whole blood staining tubes were lysed using Lysis/Fixation buffer (Biolegend) for 7 min at RT and washed with PBS 1X + 0.5% BSA and pelleted at 200 g for 7 min. Cells were then fixed with Fixation buffer (Biolegend) diluted at 1:4 in PBS 1X. Antibodies are described in Table S1.

For functional assays, 1 × 10^6^ PBMCs were incubated overnight in the presence of overlapping SIV^gag^ or SIV^nef^ peptides at a final concentration of 2 µg/mL, at 37°C and 5% CO^2^. Brefeldin A was added after 2 h (final concentration: 10 µg/mL) to inhibit cytokine secretion. At the end of the incubation, cells were washed, fixed, permeabilized (Cytofix/Cytoperm, BD), and stained. T cell responses were characterized by measuring the frequency of CD8+ T cells expressing IFN‐γ (clone B27, BD), TNF‐α (clone Mab11, Biolegend), and CD69 (clone FN50, Biolegend). The CD3 (clone SP34‐2, BD), CD4 (clone L200, Becton Dickinson), and CD8 (clone BW135/80, Miltenyi) antibodies were used as lineage markers. For the assessment of memory potential, PBMCs were labelled with CFSE at 1 µM (Invitrogen, ref C34554) and stimulated with a pool of optimal SIV peptides (2 µg/mL) for 6 days as previously described [64]. Twelve hours before completing the culture, PBMCs were re‐stimulated with the peptide pool. Brefeldin A (10 µg/mL; Invitrogen, ref 00‐4506‐51) and Monensin (1 µg/mL; BD Biosciences, ref 554724) were added at this time.

Stained‐cell suspensions were acquired with a five‐laser ZE5 cytometer (Biorad). FlowJo v10.10.0. was used for the analysis of all cytometry data.

### 4.18 Analysis of Immune Soluble Mediators and Humoral Response

Cytokine and chemokine concentrations were quantified in EDTA plasma using Non‐Human Primate Cytokine/Chemokine/Growth Factor 38‐plex milliplex kit (Merck, PRCYTA‐40K‐PX38) for sCD137, CD40L, sFASL, G‐CSF, GM‐CSF, Granzyme A, Granzyme B, IFN‐α2, IFN‐γ, IL‐1β, IL‐1RA, IL‐2, IL‐4, IL‐5, IL‐6, IL‐7, IL‐8, IL‐10, IL‐12 (p70), IL‐15, IL‐17A, IL‐18, IL‐21, IL‐22, IL‐23, IL‐33, IP‐10, I‐TAC, MCP‐1, MIG, MIP‐1α, MIP‐1β, MIP‐3α, Perforin, RANTES, TGFα, TNF‐α, VEGF‐A and a Bioplex 200 analyzer (Bio‐Rad) according to manufacturer's instructions. NHP‐anti‐virus response Legendplex kits (Biolegend) were also used. Assays were performed according to the manufacturers' recommendations. Final concentrations were obtained using the manufacturer's software.

An ELISA assay was performed using the Genscreen HIV‐1/2 Version 2 kit to assess the production of anti‐SIV antibodies.

### 4.19 SIV Viral Load Quantification

Plasma from whole blood samples was obtained by centrifugation at 1500 g for 10 min. SIV RNA was isolated from plasma using a Nucleospin 96 Virus Core kit (Macherey‐Nagel) or a QIAamp UltraSens Virus kit (Qiagen), according to manufacturer specifications. Quantitative RT‐PCR using primers and a probe targeting the *gag* region of SIV genomic RNA was used to quantify plasma viral load, as previously described [31].

### 4.20 Quantification of Intact and Defective SIV DNA Genomes

Cell‐associated SIV DNA was quantified in PBMCs, secondary lymphoid organs, including lymph nodes and spleen, using an assay designed to distinguish intact from defective SIV genomes, as previously described [65].

### 4.21 Statistical Analysis and Data Transformation

Cell count was determined using frequency among CD45^+^ cells, from whole blood cells, reported to the complete blood count of leukocytes.

Normalizations of anti‐LILRB1/B2 staining during competition assays were performed by using the formula:

$$\;x_{normalized}=\frac{x-x_{min}}{x_{max}-x_{min}}$$

All statistical analyses were performed using Prism 10 (GraphPad Software). A nonparametric Mann‐Whitney U test or a paired Wilcoxon signed‐rank test was used for unpaired or paired data, respectively. Friedman test was used to analyze longitudinal data. Comparisons of data between groups, time points, or pooled sample analyses used a Kruskal‐Wallis test and post‐hoc tests.

## Author Contributions

**Sixtine Coindre**: investigation, methodology, formal analysis, data curation. **Roger Le Grand**: validation, formal analysis, funding acquisition. **Olivier Lambotte**: validation, formal analysis, funding acquisition, writing – review and editing. **Benoit Favier**: conceptualization, funding acquisition, validation, methodology, visualization, writing – review and editing, writing – original draft, formal analysis, supervision, investigation. **Nathalie Dereuddre‐Bosquet**: investigation, validation, formal analysis. **Véronique Avettand‐Fenoel**: investigation, formal analysis. **Anne Wijkhuisen**: investigation, methodology, formal analysis, data curation. **Laurent Abi‐Rached**: investigation, writing – review and editing. **Romain Marlin**: investigation, data curation, formal analysis. **Melyssa Yaugel‐Novoa**: investigation, formal analysis. **Florian Meurisse**: investigation, writing – original draft, formal analysis, data curation, methodology, validation. **Juliette Pons**: investigation, data curation, formal analysis. **Anne‐Sophie Gallouet**: investigation, formal analysis, methodology. **Laure Fournier Le Ray**: investigation, data curation, formal analysis. **Mael Gourves**: investigation, methodology, formal analysis. **Asier Saez‐Cirion**: validation, formal analysis, writing – review and editing. **Hisashi Arase**: resources. **Gerard Zurawski**: resources, writing – review and editing. **Sandra Zurawski**: resources. **Stéphanie Simon**: validation, formal analysis. **Francis Relouzat**: investigation, methodology, validation.

## Conflicts of Interest

The authors declare no conflicts of interest.

## Supporting information




**Supporting File**: advs76557‐sup‐0001‐SuppMat.pdf.

## Data Availability

The data that support the findings of this study are available on request from the corresponding author. The data are not publicly available due to privacy or ethical restrictions.
