# Supplementary material for: Dual Blockade of LILRB1 and LILRB2 Enhances Antiviral Immune Responses in SIV Infection
Source: Adv Sci (Weinh). 2026 Jul 30:e76557. Online ahead of print. doi: 10.1002/advs.76557 (PMC13423486; doi:10.1002/advs.76557)
Supplement: Supplementary file 1 — Supporting File: advs76557‐sup‐0001‐SuppMat.pdf. [file ADVS-9999-e76557-s001.pdf]

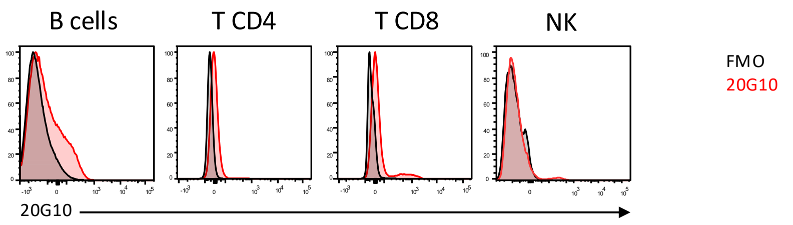

Figure S01. 20G10 mAb staining distribution on blood lymphoid cells from cynomolgus macaque.

Distribution of 20G10 mAb staining was assessed on PBMCs from cynomolgus macaque using flow cytometry.

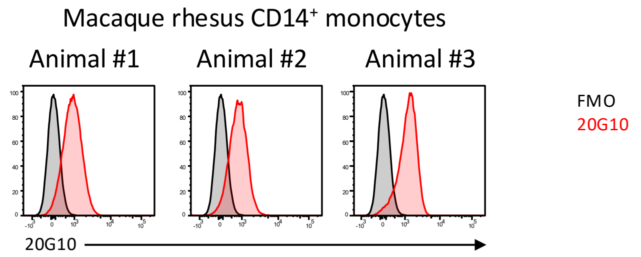

Figure S02. Cross-reactivity of 20G10 mAb with rhesus macaque.

Cross-reactivity of 20G10 mAb with rhesus macaque monocytes was assessed by flow cytometry. PBMCs from rhesus macaque were used for staining of $CD14^+$ monocytes (n=3 animals).

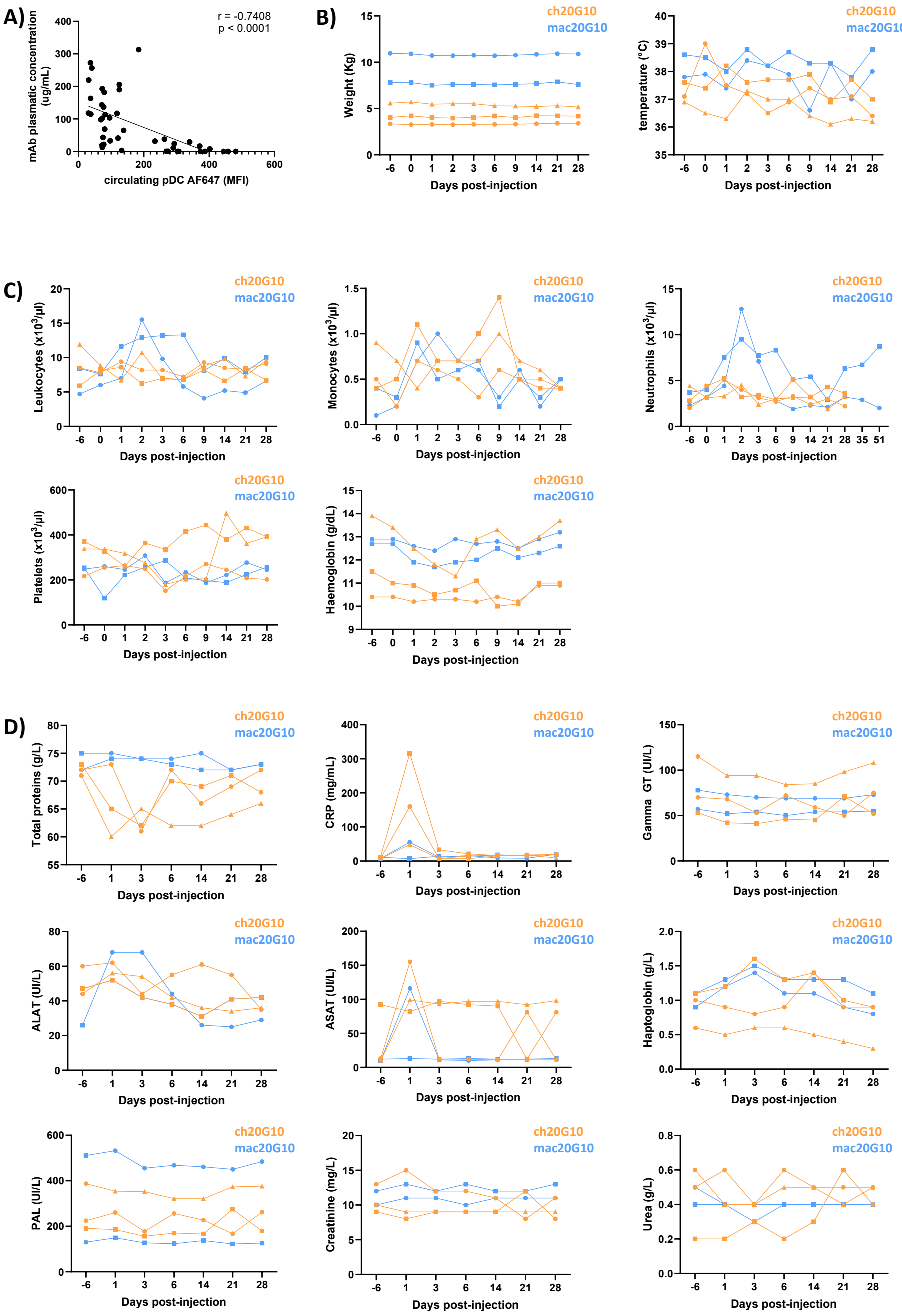

Figure S03. Longitudinal analysis of pharmacodynamic parameters following ch20G10 or mac20G10 administration in cynomolgus macaques.

A) Correlation between plasma persistence of ch20G10 and mac20G10 and competition assay performed on circulating pDCs. Non-parametric spearman correlation test was used.

B-D) Monitoring of animal weight and temperature (B), complete blood count (C) and biochemical parameters (D) after administration of ch20G10 (orange, n=3) or mac20G10 (blue, n=2).

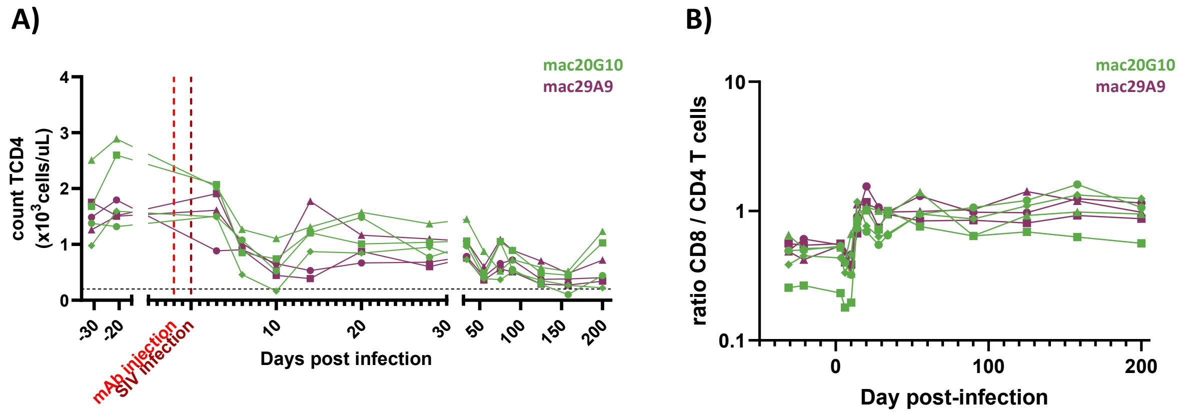

Figure S04. Follow-up of CD4⁺ T cells in SIV-infected cynomolgus macaques.

A) Monitoring of CD4⁺ T cells absolute count per μL of blood determined by flow cytometry and complete blood count.

B) Ratio of CD8⁺ T cell/CD4⁺ T cell in blood.

mac20G10 treated group is shown in green (n=4) and mac29A9 isotype control group in purple (n=3).

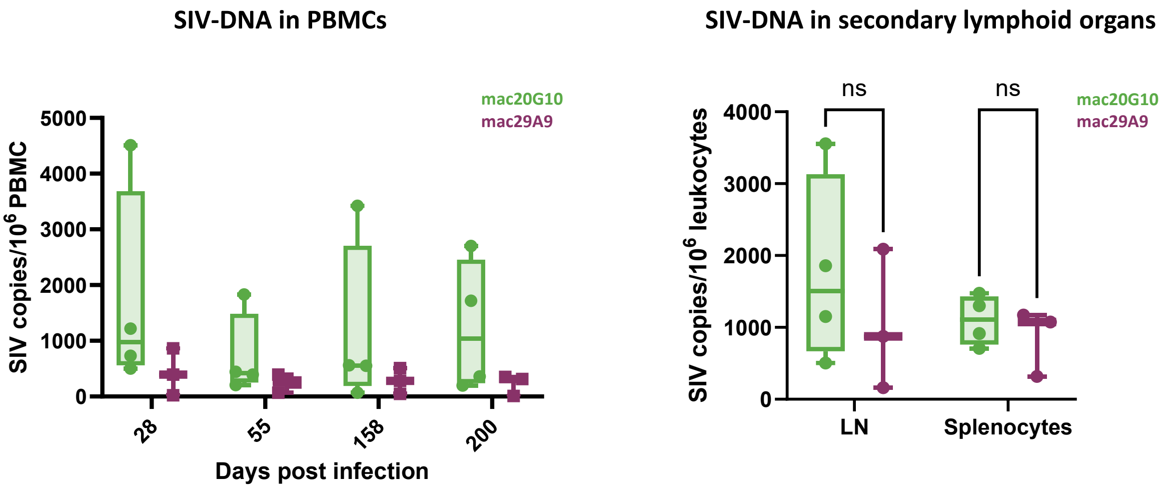

Figure S05. Kinetics of intact SIV-DNA in blood and secondary lymphoid organs.

Left panel shows the kinetics of intact SIV-DNA in PBMCs over time.

Right panel shows intact SIV-DNA assessed after euthanasia (day 200) in secondary lymphoid organs (lymph nodes and spleen).

mac20G10 treated group is shown in green (n=4) and mac29A9 isotype control group in purple (n=3). Data are represented as median with interquartile range. Groups were compared using a Wilcoxon rank-sum test.

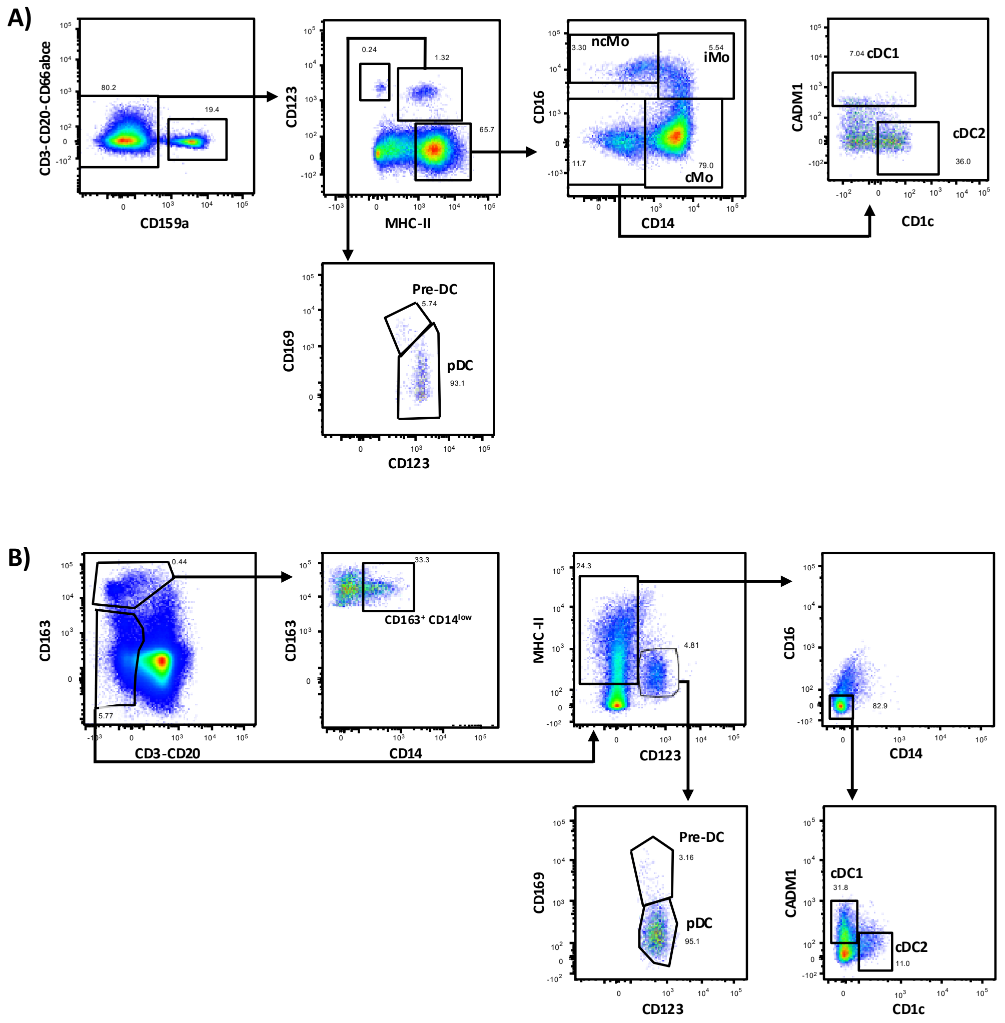

Figure S06. Flow cytometry strategy used to follow myeloid subsets in blood and lymph nodes of cynomolgus macaques.

A) Flow cytometry strategy used to isolate cDC1, cDC2, pDC and monocytes subsets in blood. cMo = classical monocytes, iMo = inflammatory monocytes and ncMo = non-classical monocytes.

B) Flow cytometry strategy used to isolate cDC1, cDC2, pDC and macrophages in lymph nodes.

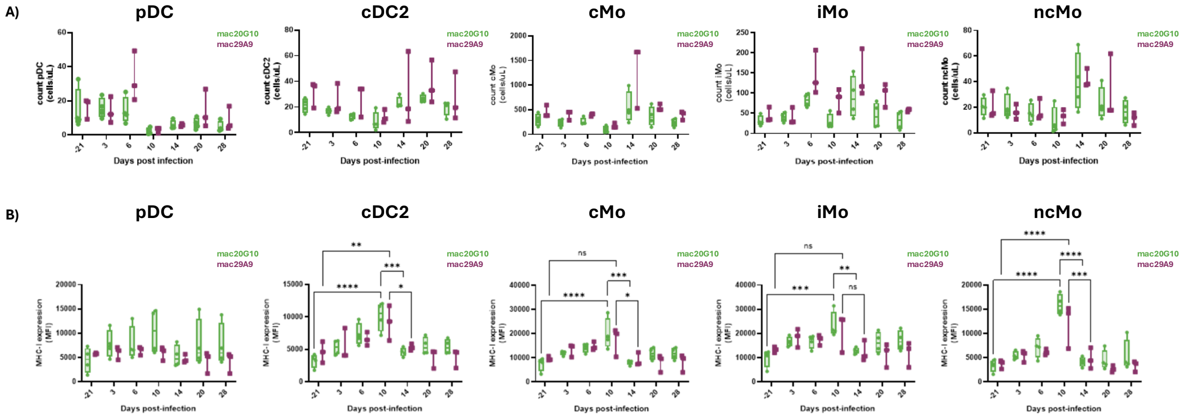

Figure S07. Follow-up of pDC, cDC2 and monocytes count and their expression of MHC-I expression in early SIV infection of macaques treated with mac20G10 or mac29A9.

A) Count of pDC, cDC2 and monocytes subsets during SIV infection. cMo = classical monocytes, iMo = inflammatory monocytes and ncMo = non-classical monocytes.

B) Evolution of MHC-I expression on pDC, cDC2 and monocytes sub-populations measured by flow cytometry. Groups and time points were compared using a Kruskal-Wallis test, followed by a Dunn's multiple comparisons test as post-hoc analysis; * = $p<0.05$, ** = $p<0.01$, *** $p<0.001$, **** $p<0.0001$.

mac20G10 treated group is shown in green (n=4) and isotype control group in purple (n=3). Data are represented as median with interquartile range.

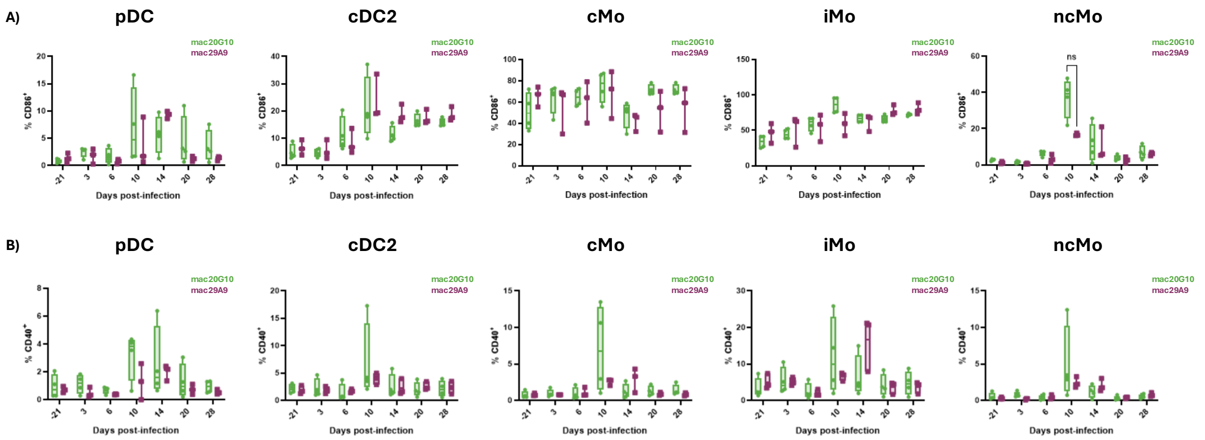

Figure S08. Follow-up of the frequency of CD86$^+$ and CD40$^+$ myeloid subsets from SIV-infected cynomolgus macaques treated with mac20G10 or mac29A9.

A) Follow up of CD86$^+$ pDC, cDC2 and monocyte sub-populations frequencies during acute phase of infection. cMo = classical monocytes, iMo = inflammatory monocytes and ncMo = non-classical monocytes. Groups and time points were compared using a Kruskal-Wallis test, followed by post-hoc analysis.

B) Follow up of the CD40$^+$ pDC, cDC2 and monocytes subpopulations frequencies during acute phase of infection. Groups and time points were compared using a Kruskal-Wallis test, followed by a Dunn's multiple comparisons test as post-hoc analysis.

mac20G10 treated group is shown in green (n=4) and isotype control group in purple (n=3). Data are represented as median with interquartile range.

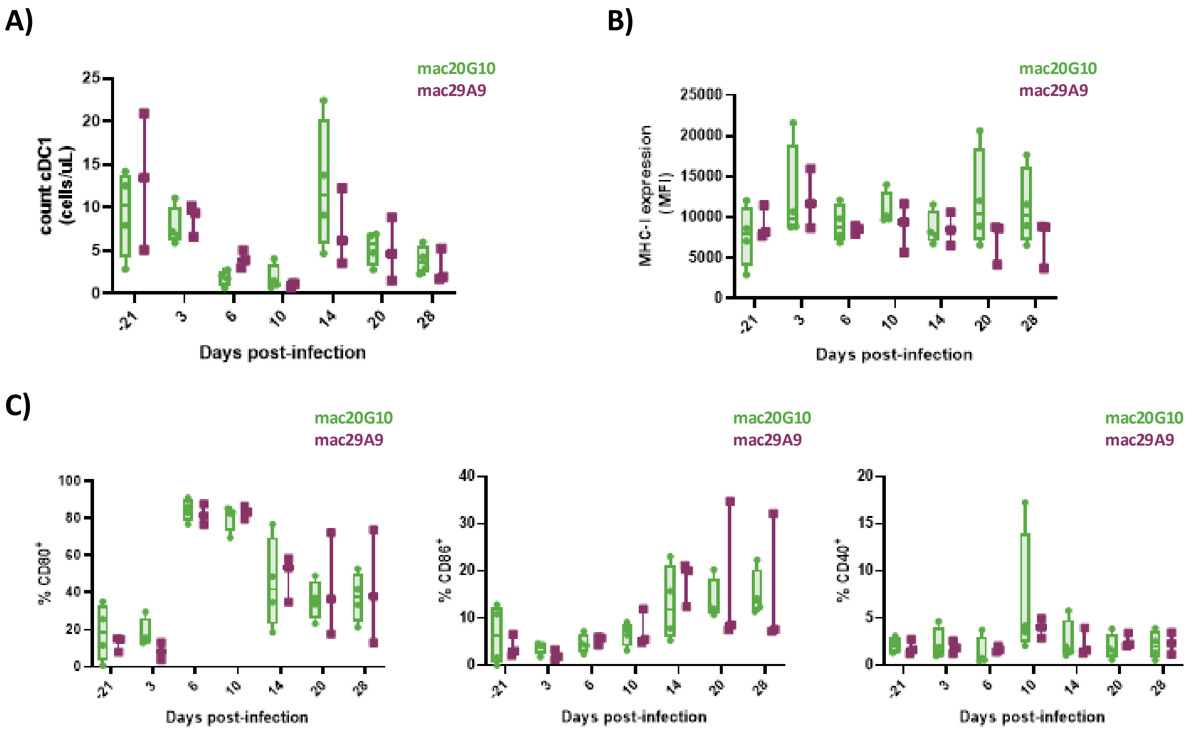

Figure S09. Follow-up of cDC1 count and phenotype from SIV-infected cynomolgus macaques treated with mac20G10 or mac29A9.

A) Count of cDC1 during SIV infection.

B) Evolution of MHC-I expression on cDC1, measured by flow cytometry.

C) Follow up of $CD80^+$, $CD86^+$ or $CD40^+$ cDC1 frequencies. Groups and time points were compared using a Kruskal-Wallis test, followed by a Dunn's multiple comparisons test as post-hoc analysis; * = p<0.05, ** = p<0.01, *** p<0.001, **** p<0.0001.

mac20G10 treated group is shown in green (n=4) and isotype control group in purple (n=3). Data are represented as median with interquartile range.

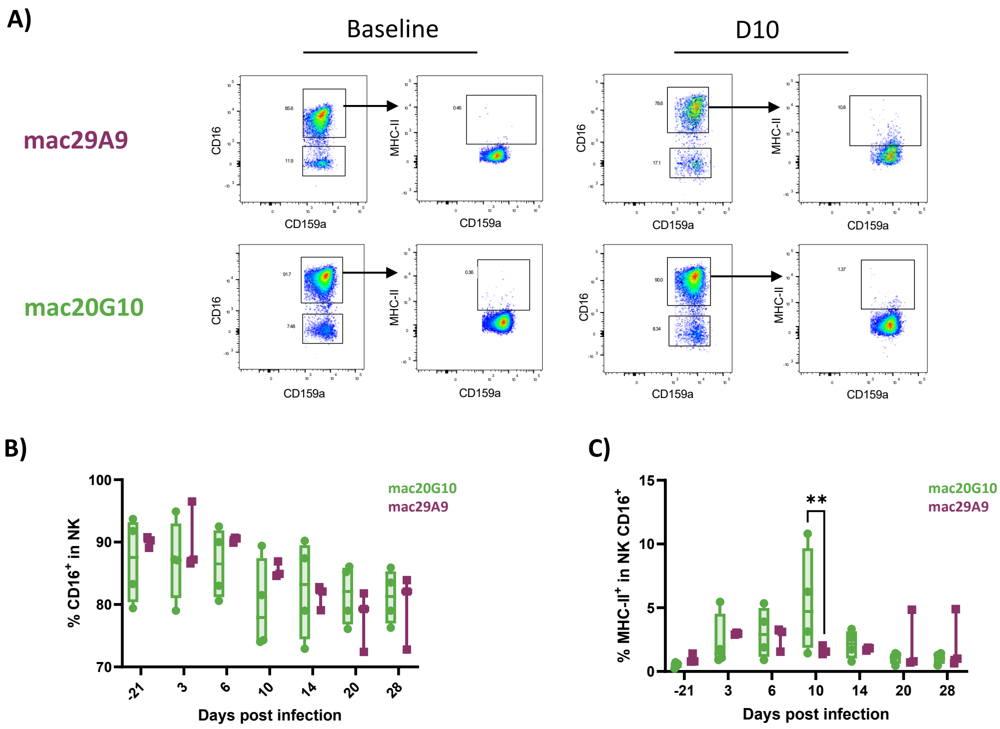

Figure S10: Kinetics of CD16^{+} NK cells population activation during acute infection by SIV under mac20G10 treatment.

A) Representative flow cytometry dot plot showing the CD16^{+} NK subset and their activation state, determined as MHC-II^{+}, during early SIV infection in cynomolgus macaques treated with mac20G10 or isotype control.

B) Follow-up by flow cytometry of CD16^{+} NK population during early SIV infection in both groups. Data are represented as median with interquartile range. Groups and time points were compared using a Kruskal-Wallis test, followed by a Dunn's multiple comparisons test as post-hoc analysis.

C) Follow-up by flow cytometry of CD16^{+} MHC-II^{+} NK population during early SIV infection in both groups. Data are represented as median with interquartile range. Groups and time points were compared using a Kruskal-Wallis test, followed by a Dunn's multiple comparisons test as post-hoc analysis; ** = $p<0.01$.

mac20G10 treated group is shown in green (n=4) and isotype control group in purple (n=3).

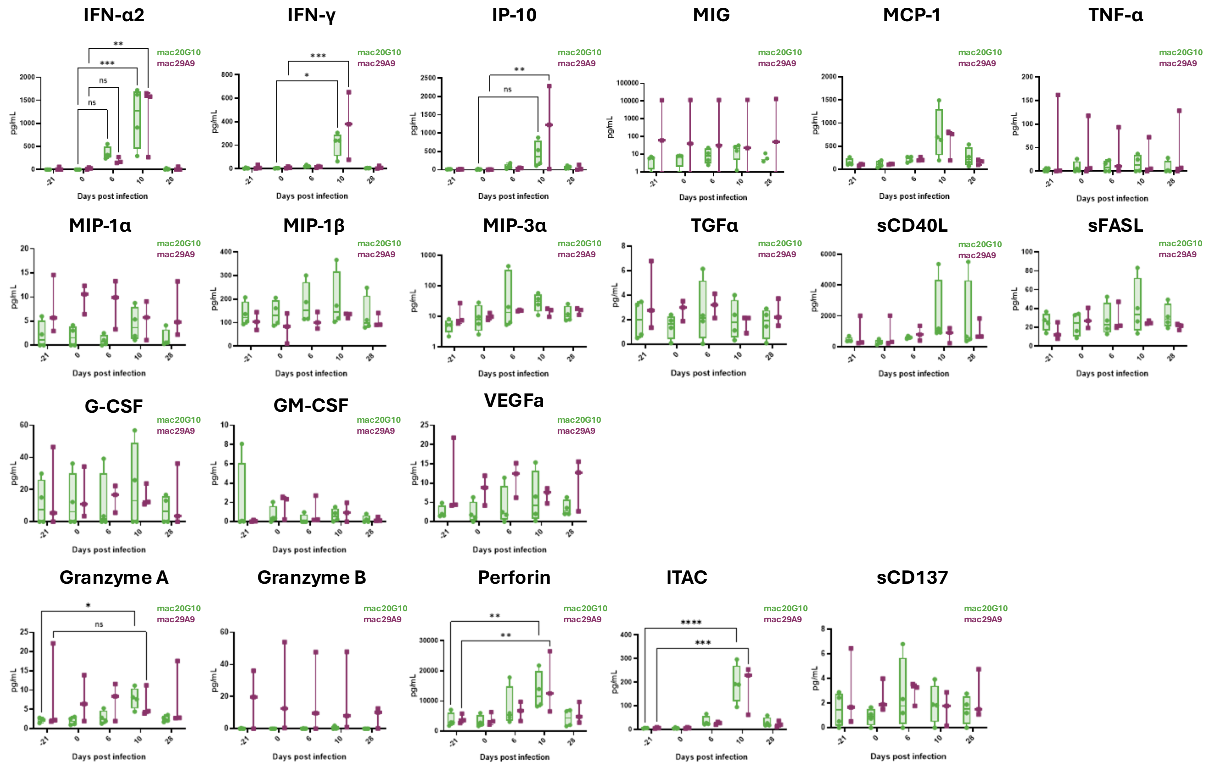

Figure S11. Follow-up of plasma cytokines in early SIV infection from cynomolgus macaques treated with mac20G10 or mac29A9.

Concentrations of IFN-α2, IFN-γ, IP-10, MIG, MIP-1α, MIP-1β, MIP-3α, MCP-1, TGFα, TNF-α, soluble CD40 ligand (sCD40L), soluble FAS ligand (sFASL), G-CSF, GM-CSF, VEGF-a, Granzyme A, Granzyme B, Perforin, ITAC and CD137 were measured using Luminex.

Groups and time points were compared using a Kruskal-Wallis test, followed by a Dunn's multiple comparisons test post-hoc analysis; * = $p<0.05$, ** = $p<0.01$, *** $p<0.001$, **** $p<0.0001$.

mac20G10 treated group is shown in green (n=4) and isotype control group in purple (n=3).

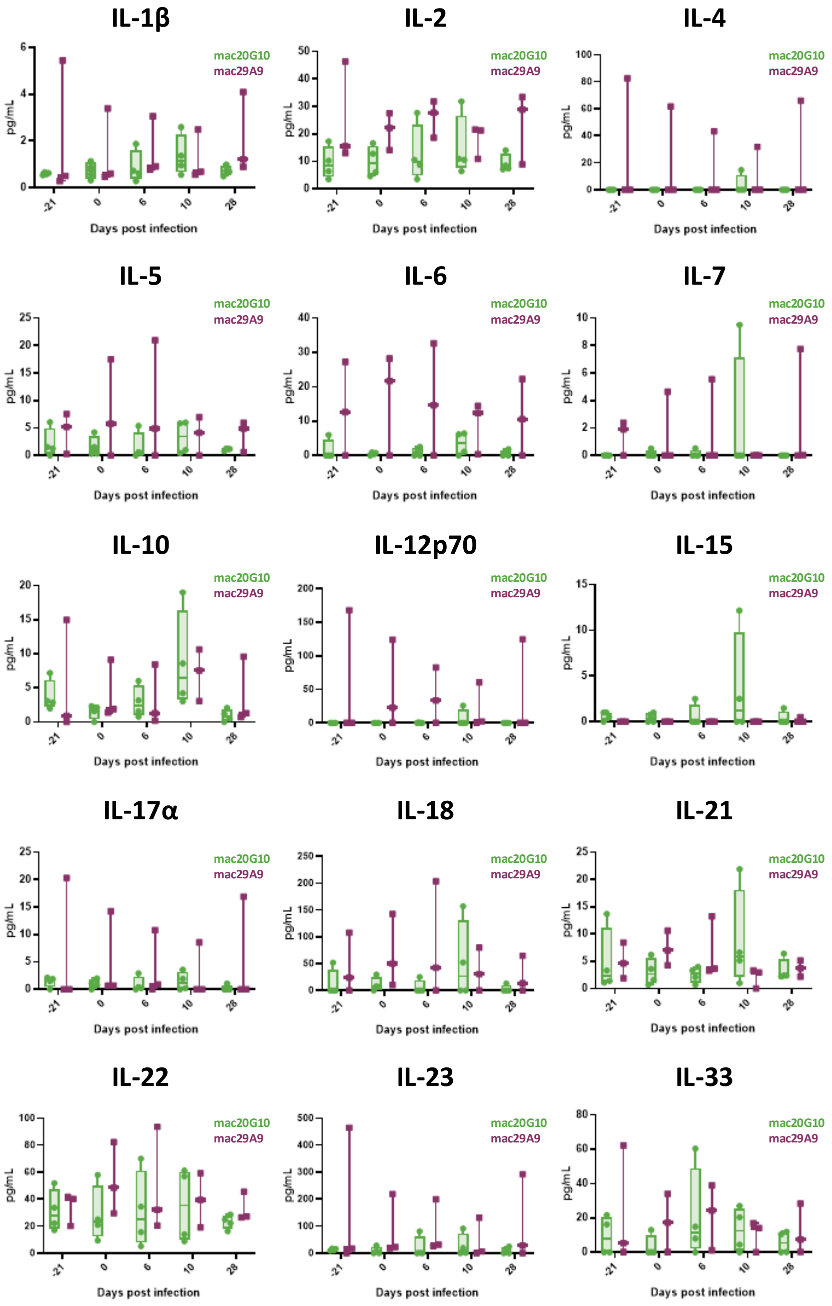

Figure S12. Follow-up of plasma interleukins in early SIV infection of cynomolgus macaques treated with mac20G10 or mac29A9.

Concentrations of IL-1β, IL-2, IL-4, IL-5, IL-6, IL-7, IL-10, IL-12p70, IL-15, IL-17α, IL-18, IL-21, IL-22, IL-23, IL-33 were measured by Luminex.

Groups and time points were compared using a Kruskal-Wallis test, followed by a Dunn's multiple comparisons test post-hoc analysis.

mac20G10 treated group is shown in green (n=4) and isotype control group in purple (n=3).

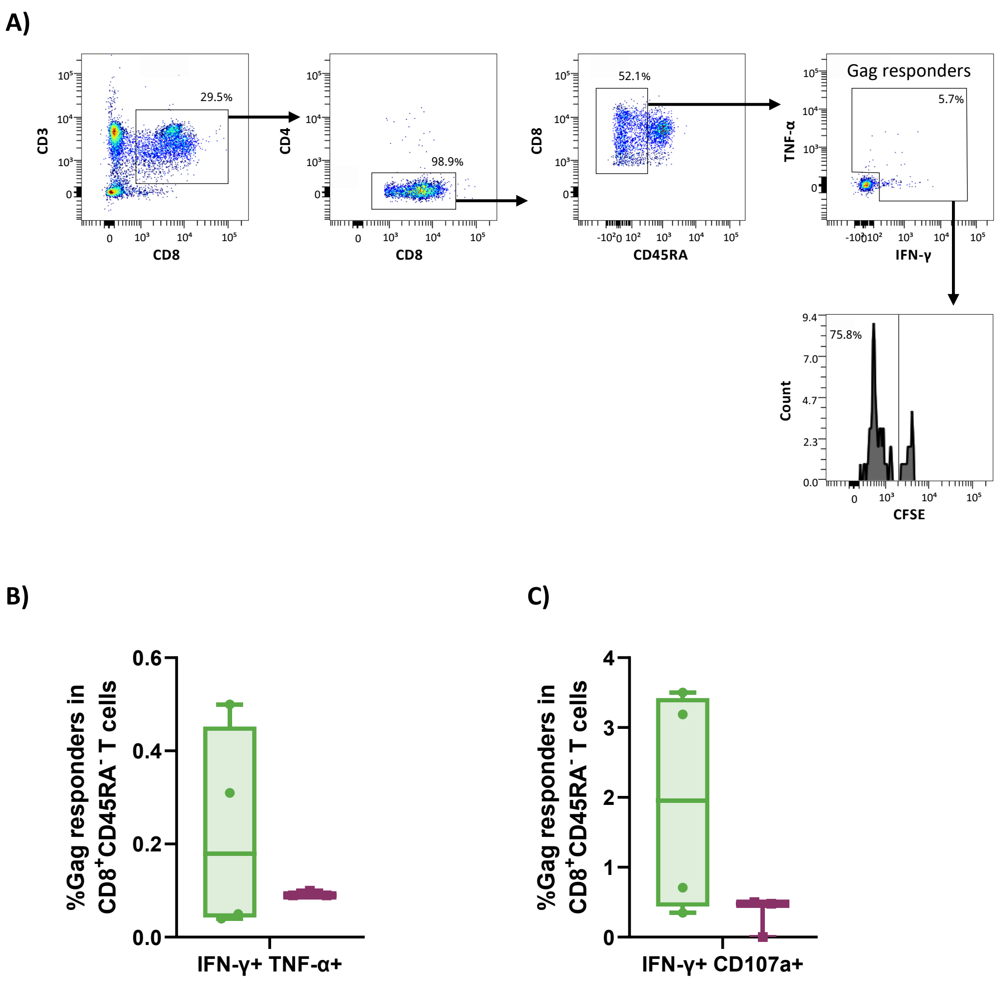

Figure S13. Evaluation of mac20G10 administration on CD8$^+$ CD45RA$^-$ T cells responses to optimized SIV gag peptide pool stimulation.

A) Flow cytometry gating strategy used for the identification of SIV^{gag} specific memory CD8$^+$ T cells after *ex vivo* peptide stimulation.

B-C) Frequency of polyfunctional IFNγ+TNFα+ (B) or IFNγ+CD107a+ (C) responses among Gag-specific CD8+ CD45RA- T cells at euthanasia (day 200). Data are represented as median with interquartile range. Groups were compared using a Wilcoxon rank-sum test. mac20G10 treated group is shown in green (n=4) and mac29A9 isotype control group in purple (n=3).

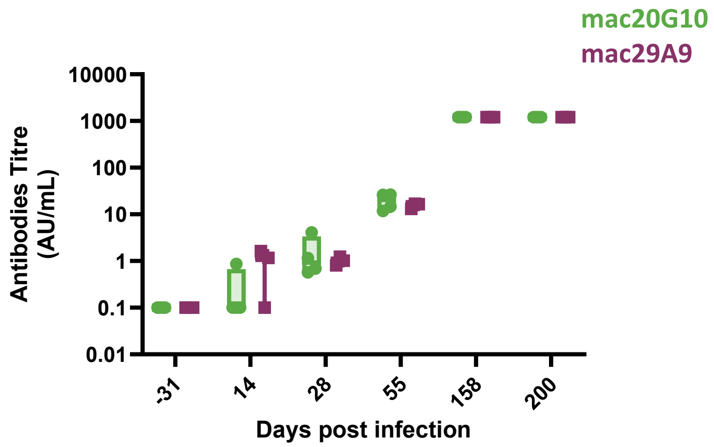

Figure S14. Follow-up of antibodies development against SIV.

A) Anti-SIV antibodies were assessed using ELISA. Antibodies titre are expressed in arbitrary units per mL (AU/mL). Values below the detection limit at baseline and day 14 are represented as 0.1 and the upper limit of detection was 1200 AU/mL.

mac20G10 treated group is shown in green (n=4) and mac29A9 isotype control group in purple (n=3). Data are represented as median with interquartile range.

Target	clone	fluorochrome	manufacturer	reference	panel
CADM1	PE	3E1	MBL	CM004-5	All + CA
CD123	PerCP-Cy5.5	7G3	BD	558714	All + CA
CD14	BV711	M5E2	Biolegend	301838	All + CA
CD159a	PE-Vio770	REA110	Miltenyi	130-113-567	Blood; SIV
CD16	BV650	3G8	Biolegend	302042	All + CA
CD163	PE-Cy7	GHI/61	Biolegend	333614	LN
CD1c	AF700	L161	Biolegend	331530	All + CA
CD20	PacificBlue	2H7	Biolegend	302328	All
CD3	Pacific Blue	SP34-2	BD	558124	All
CD40	FITC	5c3	Biolegend	334306	SIV
CD45	V500	D058-1283	BD	561489	All + CA
CD45RA	PE-Cy7	5H9	BD	561216	SIV
CD66a,b,c,e	VioBlue	TET2	Miltenyi	130-119-851	Blood; SIV
CD80	BUV395	L307.4	BD	565210	SIV
CD86	BUV737	BU63	BD	748376	SIV
CD95	BV785	DX2	Biolegend	305646	SIV
HLA-DR	BUV661	L243	BD	753687	SIV
HLA-DR	PE-Cy7	L243	BD	335795	PK + CA
HLA-I	BV605	W6/32	Biolegend	311432	SIV
Live/Dead	BlueVid		Invitrogen	L34962	All + CA
mLILRB1/B2	AF647	ch20G10; mac20G10	Fusion antibodies; biotem	homemade	All + CA
PD-L1	BV421	29E.2A3	BD	568320	SIV
Siglec1	PEdazzle594	7-239	Biolegend	346016	SIV

Supplementary table 1: Antibodies used for phenotypic panels.

Panels description: All = used for PK and SIV studies; Blood = Used only in blood panel; CA = used for cross-reactivity/cellular distribution assay; LN = Used for lymph node staining; PK = used for pharmacokinetic studies; SIV = used during SIV study.
